# Living on the edge: timing of Rand Flora disjunctions congruent with ongoing aridification in Africa

**DOI:** 10.3389/fgene.2015.00154

**Published:** 2015-05-01

**Authors:** Lisa Pokorny, Ricarda Riina, Mario Mairal, Andrea S. Meseguer, Victoria Culshaw, Jon Cendoya, Miguel Serrano, Rodrigo Carbajal, Santiago Ortiz, Myriam Heuertz, Isabel Sanmartín

**Affiliations:** ^1^Real Jardín Botánico (RJB-CSIC)Madrid, Spain; ^2^INRA, UMR 1062, Centre de Biologie pour la Gestion des Populations (INRA, IRD, CIRAD, Montpellier SupAgro)Montferrier-sur-Lez, France; ^3^Department of Botany, Pharmacy School, University of Santiago de CompostelaSantiago de Compostela, Spain; ^4^Forest Research Centre (INIA-CIFOR)Madrid, Spain; ^5^INRA, BIOGECO, UMR 1202Cestas, France; ^6^University of Bordeaux, BIOGECO, UMR 1202Talence, France

**Keywords:** Africa, historical biogeography, climate change, diversification rates, long-distance dispersal, Rand Flora, vicariance

## Abstract

The Rand Flora is a well-known floristic pattern in which unrelated plant lineages show similar disjunct distributions in the continental margins of Africa and adjacent islands—Macaronesia-northwest Africa, Horn of Africa-Southern Arabia, Eastern Africa, and Southern Africa. These lineages are now separated by environmental barriers such as the arid regions of the Sahara and Kalahari Deserts or the tropical lowlands of Central Africa. Alternative explanations for the Rand Flora pattern range from vicariance and climate-driven extinction of a widespread pan-African flora to independent dispersal events and speciation *in situ*. To provide a temporal framework for this pattern, we used published data from nuclear and chloroplast DNA to estimate the age of disjunction of 17 lineages that span 12 families and nine orders of angiosperms. We further used these estimates to infer diversification rates for Rand Flora disjunct clades in relation to their higher-level encompassing lineages. Our results indicate that most disjunctions fall within the Miocene and Pliocene periods, coinciding with the onset of a major aridification trend, still ongoing, in Africa. Age of disjunctions seemed to be related to the climatic affinities of each Rand Flora lineage, with sub-humid taxa dated earlier (e.g., *Sideroxylon*) and those with more xeric affinities (e.g., *Campylanthus*) diverging later. We did not find support for significant decreases in diversification rates in most groups, with the exception of older subtropical lineages (e.g., *Sideroxylon*, *Hypericum*, or *Canarina*), but some lineages (e.g., *Cicer*, *Campylanthus*) showed a long temporal gap between stem and crown ages, suggestive of extinction. In all, the Rand Flora pattern seems to fit the definition of biogeographic pseudocongruence, with the pattern arising at different times in response to the increasing aridity of the African continent, with interspersed periods of humidity allowing range expansions.

## Introduction

Large-scale biodiversity patterns have intrigued naturalists since the eighteenth century (Forster, 1778; von Humboldt and Bonpland, 1805; Wallace, 1878; Fischer, 1960; Stevens, 1989; Lomolino et al., 2010). Recognizing that spatial variation in environmental variables such as temperature or precipitation is insufficient to explain such patterns, more integrative explanations that emphasize the role of both environmental and evolutionary factors have recently been advanced (Qian and Ricklefs, 2000; Wiens and Donoghue, 2004; Jablonski et al., 2006). As Wiens and Donoghue (2004) state “environmental variables cannot by themselves increase or decrease local or regional species richness”; only evolutionary processes such as dispersal, speciation and extinction can. Therefore, reconstructing rates of dispersal, speciation, and extinction across the component lineages of a biota might help us understand how assembly took place across space and through time (Pennington et al., 2004; Ricklefs, 2007; Wiens, 2011). Moreover, understanding patterns of biotic assembly is a pressing goal in biodiversity research at a time when nearly one tenth of species on Earth are projected to disappear in the next hundred years (Maclean and Wilson, 2011).

**Figure 1:**
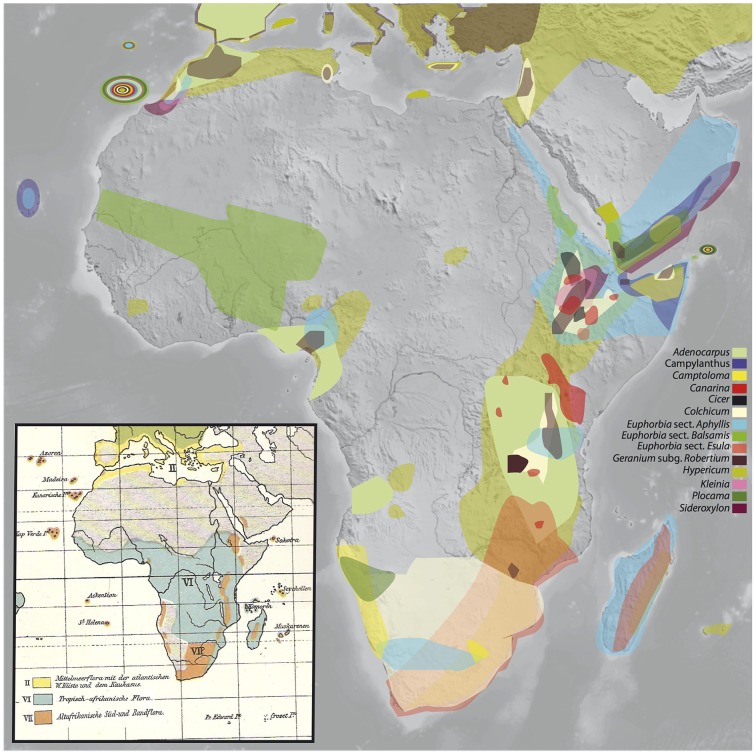
**Rand Flora disjunction pattern as evidenced by angiosperm plant lineages analyzed for this study**. The inset shows K.H.H. Christ's (1910) depiction of “cette flore *marginale* de l'Afrique” or “Randflora” (in orange color), note their similar geographic limits. Taxa: *Adenocarpus* (Fabaceae), *Camptoloma* (Scrophulariaceae), *Campylanthus* (Plantaginaceae), *Canarina* (Platycodoneae, Campanulaceae), *Cicer* (Fabaceae), *Colchicum* (Colchicaceae), *Euphorbia* subgen. *Athymalus* (sects. *Anthacanthae* and *Balsamis*; Euphorbiaceae), *Euphorbia* subgen. *Esula* (sect. *Aphyllis*), *Euphorbia* subgen. *Esula* (African clade of sect. Esula), *Geranium* subgen. *Robertium* (Geraniaceae), *Hypericum* (Hypericaceae), *Kleinia* (Asteraceae), *Plocama* (Rubiaceae), and *Sideroxylon* (Sideroxyleae, Sapotaceae).

Africa is a continent especially interesting to study patterns of biotic assembly. On one hand, African tropical regions are comparatively species-poorer than regions situated in the same equatorial latitudes in the Neotropics and Southeast Asia (Lavin et al., 2001; Couvreur, 2015), which has led to the continent being referred to as the “odd man out” (Richards, 1973). On the other, Africa offers some extraordinary examples of continent-wide disjunctions. For example, tropical rainforests in Africa appear in two main blocks, the West-Central Guineo-Congolian region and the coastal and montane regions of East Africa, now separated by a 1000 Km-wide arid corridor (Couvreur et al., 2008). Another prime example is the so called *Rand Flora* (RF), a biogeographic pattern in which unrelated plant lineages show comparable disjunct distributions with sister taxa occurring on now distantly located regions in the continental margins of Africa: Macaronesia-northwest Africa, Western African mountains, Horn of Africa-South Arabia (including the Island of Socotra), Eastern Africa (incl. Madagascar), and Southern Africa (Christ, 1892; Lebrun, 1947, 1961; Quézel, 1978; Andrus et al., 2004; Sanmartín et al., 2010; Figure 1). All RF lineages share sub-humid to xerophilic affinities, so that the tropical lowlands of Central Africa and the large Sahara and Arabian deserts in the north or the Namib and Kalahari deserts in the south presumably constitute effective climatic barriers to their dispersal.

Swiss botanist K. H. H. Christ (1892) first referred to “cette flore *marginale* de l'Afrique,” that is “this *marginal* African flora,” in a note addressing the role the so called *ancient African flora* played on European floras, with emphasis on the Mediterranean biome. Later, in his “Die Geographie der Farne” (i.e., “The Geography of Ferns”; Christ, 1910), he very aptly named this geographic pattern “Randflora” (see pp. 259–275), where the Germanic word “Rand” stands for rim, edge, border, margin (see Figure 1 inset), noting its similarities with Engler's “afrikanisch-makaronesische Element” (Engler, 1879, 1910; see pp. 76 in the former and pp. 983–984 and 1010 in the latter), that is, an “Afro-Macaronesian element” linking disjunct xerophilic taxa found in the continental margins of Africa and its adjacent islands (e.g., Canary Islands, Cape Verde, etc.).

Historical explanations for this pattern and, in particular, its temporal framework, its exact boundaries, and the ecology of the plants involved have varied through these past two centuries. The early view (Engler, 1879, 1910; Christ, 1892, 1910) was one of a pan-African flora found throughout the continent that became restricted to its margins as a result of major climate changes (i.e., increasing aridification) throughout the *Tertiary* (i.e., the Cenozoic Period, 66.0–2.58 Ma). Lebrun (1947; see pp. 134–137), and later Monod (1971, p. 377) and Quézel (1978, p. 511), interpreted Christ's *ancient African flora* as a complex ensemble that had experienced alternating expansions and contractions through time, having had a chance to spread across northern Africa during favorable moments in the Miocene and needing to retract at the end of the Neogene (i.e., Pliocene): a further increase in aridity at the beginning of Pleistocene glaciations would have confined relictual or vicariant taxa to Macaronesia, northwest Africa and Arabia. Axelrod and Raven (1978) explained some of these disjunctions in relation to a more ancient, widespread Paleogene flora of subtropical origin that covered the entire African continent at the beginning of the Cenozoic, and that was decimated by successive events of aridification, of which the relict floras of Macaronesia, the Cape Region, and the Afromontane forests in eastern and western Africa would be remnants. Bramwell (1985) explains this pattern in terms of pan-biogeographic “general tracks” that connect what would be the remains of an ancient flora that extended across the Mediterranean and Northern Africa in the Miocene, and whose vestiges could be found in the Macaronesian laurisilva and a few enclaves in the island of Socotra, the Ethiopian Highlands and southern Yemen.

These authors share a vicariant perspective and presume RF lineages were part of a widespread pan-African *Tertiary* flora that became fragmented by the appearance of climatic barriers (i.e., aridification), leaving relictual lineages with reduced distributions at “refugia” in the margins of Africa (i.e., “continental” islands). This “refugium” idea rests on the fact that many of these RF regions—Macaronesia, the South African Cape region, and the semi-arid regions of Eastern Africa and Southern Arabia (e.g., Ethiopia, Yemen, Socotra)—harbor a large number of endemic species, when compared to neighboring areas. Moreover, the “fragmentation-refugium” hypothesis implies the disappearance, possibly by extinction, of RF lineages from part of their distributional range (e.g., across the Sahara in central Northern Africa), which is consonant with the “climatic vicariance” concept (Wiens, 2004): an environmental change creates conditions within a species' geographic range that are outside the ancestral climatic tolerances; individuals are unable to persist and the species' geographic range becomes fragmented.

The alternative explanation is one of independent dispersal (immigration) events among geographically isolated regions and subsequent speciation *in situ*. In this framework, divergence events need not be congruent across lineages, since long-distance dispersal (LDD) events are highly stochastic in nature (Nathan, 2006). Asides from transoceanic dispersal—which has been postulated in the case of *Aeonium* (Kim et al., 2008), *Geranium* (Fiz et al., 2008), and other RF lineages (Andrus et al., 2004) based on molecular phylogenetic evidence—, cross-continent LDD dispersal is also possible: published examples favoring cross-continent LDD include *Senecio*, with a disjunct distribution between Macaronesia-Northern Africa and South Africa (Coleman et al., 2003; Pelser et al., 2012). Moreover, dispersal does not necessarily imply long-distance migration events. In some cases, dispersal across intermediate areas that act as “stepping stones” or “land bridges” could have been possible. For example, the presence of isolated mountain ranges (offering suitable habitats) throughout the Sahara, such as the Tibesti and Hoggar massifs, could have allowed this short or medium-range dispersal in *Campanula* (Alarcón et al., pers. comm.). Correspondingly, some RF lineages might have used the Arabian Plate as a land bridge to reach East Africa (*Campanula*, Roquet et al., 2009; *Hypericum*, Meseguer et al., 2013), and others may have benefited from the new habitats offered by the Pliocene uplift of the Eastern Arc Mountains to migrate to or from South Africa (Meseguer et al., 2013).

Discriminating between climate-driven vicariance vs. independent dispersal events between geographically isolated regions requires framing the evolution of disjunct lineages on a temporal scale (Sanmartín, 2014). On the other hand, to unravel the origin of a biota or biome, a meta-analysis across dated phylogenies of multiple non-nested clades is needed (Pennington et al., 2010; Wiens, 2011; Couvreur, 2015). Sanmartín et al. (2010) carried out a meta-analysis of 13 lineages to infer relative rates of historical dispersal among RF regions (Macaronesia, Eastern Africa-Southern Arabia, and Southern Africa) and found the highest rate of biotic exchange between east and west Northern Africa, across the Sahara. However, they did not integrate absolute estimates of lineage divergences in their inference, since very few RF lineages (e.g., Roquet et al., 2009) had been dated at the time.

In this study, we estimate time divergences for up to 13 plant lineages (Table 1) displaying RF disjunct distributions (Figure 1), and use published divergence times for four other lineages (see Materials and Methods), in order to provide a much-needed temporal framework for this pattern. An extensive description of each of these lineages, geographic distributions and phylogenetic relationships is provided in Supplementary Materials. We also frame these disjunctions in the context of major climatic and geological events in the history of Africa (see summary below) and estimate net diversification rates in an attempt to address the role that evolutionary processes, such as climate-driven extinction, may have played in the formation of the African RF pattern.

**Table 1:** **Rand Flora disjunctions, encompassing (higher level) lineages, recent molecular phylogenetic studies, and molecular markers used in here**.

**Order**	**Family**	**Tribe (or else)**	**Genus**	**Subgenus**	**Section (or else)**	**Disjunction name**	**Dataset reference**	**Molecular marker**	
								**Nuclear**	**Chloroplast**
Fabales	Fabaceae	Genisteae	*Adenocarpus*			*Ad. manii*	Cubas et al., 2010	ETS, ITS	*trn*LF
Saxifragales	Crassulaceae	*Aeonium* alliance	*Aeonium*			*Ae. leucoblepharum*	Mort et al., 2002, 2007	ITS	–
Malpighiales	Euphorbiaceae		*Euphorbia*	*Athymalus*	*Anthacanthae* *Balsamis*	*Eu. omariana* *Eu. balsamifera*	Peirson et al., 2013	ITS	*ndh*F
Malpighiales	Euphorbiaceae			*Esula*	*Aphyllis* *Esula*	*Eu. tuckeyana* *Eu. usambarica* *Eu. schimperiana*	Barres et al., 2011; Riina et al., 2013	ITS	*ndh*F
Asterales	Campanulaceae		*Campanula*		*Azorina* (clade)	*Ca. jacobaea*	Alarcón et al., 2013	–	*trn*LF, *pet*BD, *rpl*32–*trn*L, *trn*SG
Lamiales	Scrophulariaceae	Buddlejoideae (subfamily)	*Camptoloma*			*Cm. canariense* *Cm. rotundifolium*	Kornhall et al., 2001; Oxelman et al., 2005	–	*trn*LF, *ndh*F, *rps*16
Lamiales	Plantaginaceae	Globularieae	*Campylanthus*			*Cy. salsoloides*	Thiv et al., 2010	ITS	*atp*B-*rbc*L
Asterales	Campanulaceae	Platycodoneae	*Canarina*			*Cn. canariensis*	Mairal et al., 2015	ITS	*pet*BD, *psb*J, *trn*LF, *trn*SG
Fabales	Fabaceae	Vicioids (clade)	*Cicer*			*Ci. canariense*	Javadi et al., 2007	ETS, ITS	*trn*SG, *mat*K, *trn*AH, *trn*A-Leu
Liliales	Colchicaceae	Colchiceae	*Colchicum*			*Co. schimperianum*	Manning et al., 2007; del Hoyo et al., 2009	–	*trn*LF, *atp*B-*rbc*L, *rps*16
Geraniales	Geraniaceae		*Geranium*	*Robertium*		*G. robertianum*	Fiz et al., 2008	ITS	–
Malpighiales	Hypericaceae	Hypericeae	*Hypericum*		*Androsaemum* *Campylosporus*	*H. scopulorum* *H. quartinianum*	Meseguer et al., 2013	–	*trn*LF, *trn*SG
Asterlaes	Asteraceae	Senecioneae	*Kleinia*			*K. neriifolia*	Pelser et al., 2007	ITS	*trn*LF
Gentianales	Rubiaceae	Putorieae	*Plocama*			*Pl. pendula* *Pl. crocyllis*	Backlund et al., 2007	–	*rps*16, *trn*TF, *atp*B-*rbc*L
Ericales	Sapotaceae	Sideroxyleae	*Sideroxylon*			*S. spinosus*	Smedmark et al., 2006; Smedmark and Anderberg, 2007	–	*ndh*F, *trn*H–*psb*A, *trn*CD

GenBank numbers can be found in the references listed under column “Dataset reference.”

## Materials and methods

### Study area: African climate through time

To understand biogeographic patterns in the African flora, it is necessary to briefly review the climatic and geological history that might have influenced the evolution of African plant lineages. Extensive reviews of African climatic and vegetation history can be found in Axelrod and Raven (1978); van Zinderen Bakker (1978); Maley (1996, 2000); Morley (2000); Jacobs et al. (2010), Plana (2004), and Bonnefille (2011), among others.

**Figure 2:**
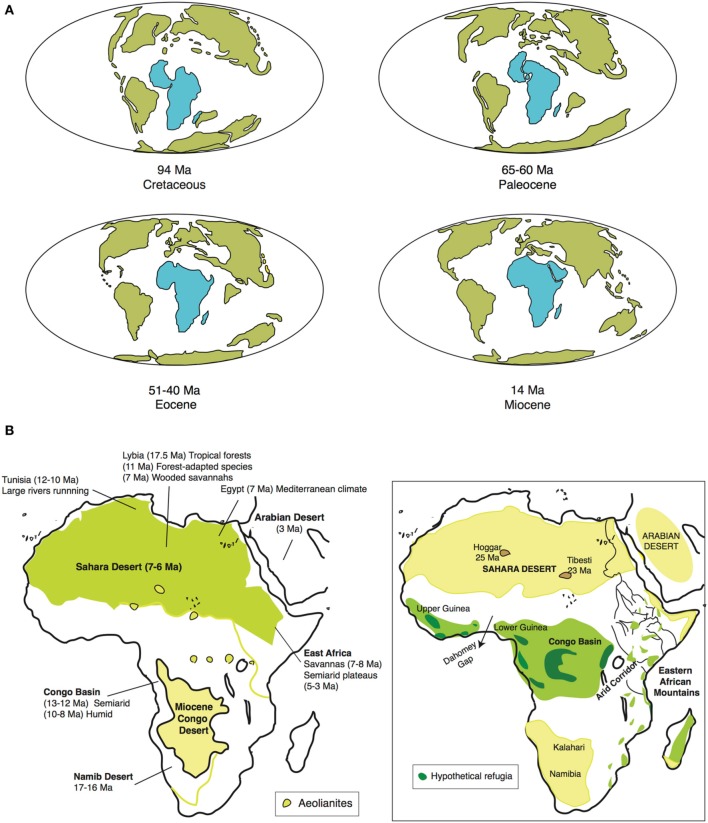
**(A)** Tectonic fragmentation of the supercontinent Gondwana through time, showing Africa's drift northwards; and **(B)** main climatic events in Africa during Neogene (adapted from Senut et al., 2009): (B-left) Early Neogene Central Africa was more arid than North Africa, with a desert, semiarid region in the Congo Basin. Desertification started in southwest Africa in the Mid-Miocene, proceeding eastward and northward, and finalizing with the formation of the Sahara Desert. Conversely, Central Africa became tropical due to subsidence and Eastern African uplift. (B-right) Schematic representation of present-day vegetation belts, showing position of main deserts and rainforest refugia (Eastern Arc Mountains/Guineo-Congolian region (the latter fragmented into smaller refugia). Rand Flora lineages occupy the regions in the margin that are not deserts or rainforests, rarely some find refuge in mountain areas of North African Sahara (e.g., Tibesti and Hoggar Massifs).

During the Late Mesozoic, Africa was part of the supercontinent Gondwana, located in the southern hemisphere, and enjoyed a relatively humid and temperate climate (Raven and Axelrod, 1974). After breaking up from South America ca. 95 Ma, Africa started moving northwards toward the equatorial zone (Figure 2A). The result was a general trend toward continental aridification in which different regions became arid or wet at alternative times (Figure 2B, Senut et al., 2009). Paleocene Africa (66–56 Ma) was mainly wet and warm, characterized by a major diversification in the West African flora (Plana, 2004). A global increase in temperatures in the Eocene (56–33.9 Ma) led to increased aridity in Central Africa, with a rainforest-savannah mosaic in the Congo region. This was followed by a global cooling event at the Eocene-Oligocene boundary (33.9 Ma), which led again to aridification and major extinction but did not change biome composition (Axelrod and Raven, 1978).

The Early Miocene (23–16 Ma) was warm and humid, with wide extension of rainforests, from the northern Sahara to parts of Southern Africa. The Mid Miocene (16–11.6 Ma) was a period of major changes in climate and topography. A combination of factors, including the gradual uplift of Eastern Africa, the successive closure of the Tethys seaway in the north, and the expansion of the East Antarctic ice sheet in the south (Trauth et al., 2009), led to a general intensification of the aridification process, though it was not homogeneous across the continent. Geological and paleontological evidence suggest that now arid regions (e.g., northern Africa, Horn of Africa, Namib Desert) were during this period more humid than they are today, whereas other now humid regions (e.g., Congo Basin) were much drier (Figure 2B). Desertification started in the southwest (Namib Desert) around 17–16 Ma ago, and proceeded eastward and northward. In Southern Africa, tropical to subtropical vegetation was replaced by wooded savannah during the lower Mid-Miocene (Senut et al., 2009). In Northern Africa, the earliest evidence of aridity in the Sahara region is from the Late Miocene (11.6–5.3 Ma), ca. 7–6 Ma (Senut et al., 2009; Figure 2B). In Central Africa, a semiarid desert (“Miocene Congo Desert,” Figure 2B) occupied the region until the Mid Miocene, 13–12 Ma ago, when the Eastern African uplift and subsequent subsidence led to the establishment of the Congo River drainage and a general increase in humidity (“tropicalization”). Also in the Late Miocene, ca. 7–8 Ma, a new period of tectonic activity in Eastern Africa led to the uplift of the Eastern Arc Mountains and the uplands of West Central Africa (Cameroon volcanic line), which led to increasing aridity and the expansion of savannahs and grasslands in these regions (Sepulchre et al., 2006). Uplifting reached a maximum during the Plio-Pleistocene and led to the formation of the Ethiopian Highlands and the desertification of low-lying areas in the Horn of Africa (Senut et al., 2009). From the Late Pliocene to the Holocene, the alternation of glacial-and interglacial periods seems to have led to repeated contractions and expansions of distributional ranges across both subtropical and tropical taxa (Maley, 2000; Bonnefille, 2011). Some areas like the Saharan massifs of Tibesti and Hoggar or the Ennedi Mountains could have served as refuges during arid periods for subtropical taxa (Osborne et al., 2008), whereas the uplands of Upper and Lower Guinea and the east of the Congo Basin, the Albertine Rift, or the Eastern Arc Mountains could have played the same role for tropical plant taxa (Maley, 1996; Figure 2B).

### Taxon sampling

**Figure 3:**
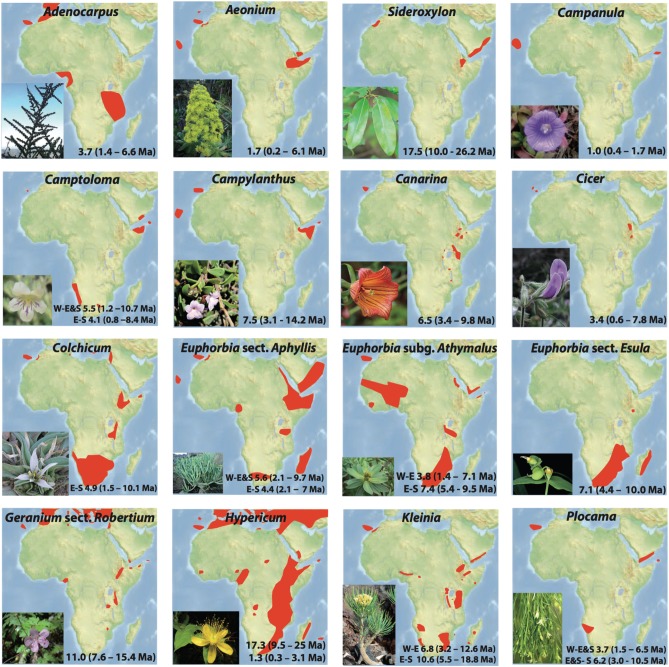
**Individual distributions and habit illustrations for 16 plant lineages exhibiting Rand Flora disjunctions**. Estimated divergence times within each lineage correspond to the disjunctions represented in **Figures 4, 5** and indicated in the MCC chronograms shown in Figures S1–S16. Taxa names correspond to those in Table 1.

We retrieved sequences from GenBank from existing studies (Table 1) for the following 13 lineages exhibiting a distribution congruent with the RF pattern (Andrus et al., 2004; Sanmartín et al., 2010): *Adenocarpus*, *Aeonium*, *Camptoloma*, *Campylanthus*, *Cicer*, *Colchicum*, *Euphorbia* sects. *Antachanthae*, *Aphyllis*, *Balsamis*, and *Esula*, *Geranium*, *Kleinia*, and *Plocama* (Figure 3). We chose these lineages because sampling is nearly complete in most cases with very few to no missing taxa. Most of these RF taxa have been sequenced for several markers from the nuclear and chloroplast DNA regions. For each group we selected the markers with most sequences and tried representing both genomic compartments whenever possible. The sequences were aligned using the Opalescent package (Opal v2.1.0; Wheeler and Kececioglu, 2007) in Mesquite v3.01 (Maddison and Maddison, 2014) and manually adjusted in SE-AL v2.0a11 (Rambaut, 2002) using a similarity criterion, as recommended by Simmons (2004). For four other RF lineages —*Campanula* (Alarcón et al., 2013), *Canarina* (Mairal et al., 2015), *Hypericum* (Meseguer et al., 2013), and *Sideroxylon* (Stride et al., 2014)—we used recently published time estimates by our research team (except for *Sideroxylon*, which nonetheless used a dating approach similar to ours). Approximately 1600 sequences from ca. 675 taxa from 12 families and 9 orders of angiosperms were included in our study (Table 1).

### Estimating absolute divergence times

Divergence times were estimated under a Bayesian framework in BEAST v1.8 (Drummond et al., 2012). For each lineage, we constructed a dataset including the markers listed in Table 1, which were partitioned by genome (chloroplast vs. nuclear), whenever possible. The best-fitting substitution model for each partition was selected using the Akaike Information Criterion implemented in MrModeltest v2.2 (Nylander, 2004) and run in PAUP^*^ v4.0b (Swofford, 2002). The relaxed uncorrelated lognormal clock model (UCLD, Drummond et al., 2006) and a Yule speciation process as tree model were selected for all datasets based on preliminary explorations. MCMC searches were run 5 × 10^7^ generations and sampled and logged every 2500th generation. We used Tracer v1.6 (Rambaut et al., 2013) to determine stationarity of the Markov chain and to verify that all parameters had large enough effective sampling sizes (ESS>200). TreeAnnotator v1.8.0 (Drummond et al., 2012) and FigTree v. 1.4.2 (Rambaut, 2009) were used respectively to generate and visualize the resulting maximum clade credibility (MCC) chronograms.

Calibration points for obtaining absolute divergence times were based on either the fossil record or on published secondary calibration constraints (Table 2). The latter were obtained from published dated phylogenies of datasets including our study groups (e.g., the family to which the genus belongs), and were assigned normal distribution priors (Ho and Phillips, 2009) in the BEAST analysis that encompassed the mean and the 95% highest posterior density (HPD) confidence interval (CI) from these studies [except in the case of time constrains from Bell et al. (2010), for which a lognormal distribution was used, since posterior estimates for a normal prior were not available]. For fossil calibration points we used a lognormal prior, since this distribution better represents the stratigraphic uncertainty associated to the fossil record (Ho and Phillips, 2009). The offset of the lognormal distribution was set to the upper bound of the stratigraphic period where the fossil was found, and the standard deviation (SD) and mean were set so that the 95% CI encompassed the lower and upper bound of the period (e.g., for Late Eocene *Hypericum antiquum* a lognormal distribution offset at 33.9 Myr, with mean = 1.0 and SD = 0.7, was used to cover the length of the period where the fossil was found, that is 33.9–37.2 Ma). A summary of time constraints used for each dataset and their provenance can be found in Table 2.

**Table 2:** **Time constraints and prior probability distributions imposed on constrained nodes to estimate divergence times in RF lineages**.

**Taxon set**	**Node constrained**	**Time constraint (Myr)**			**Dating reference**	**Figure/Table/P**.
		**Distribution (offset)**	**Mean**	**SD**		
*Adenocarpus*	ROOT: Genisteae	Normal	19.5	3.8	Lavin et al., 2005	Table 2, node 32
*Aeonium* alliance	ROOT: *Aeonium* alliance	Normal	18.83	1.0	Kim et al., 2008	Figure 2C
*E*. subg. *Athymalus*	*Athymalus* w/o *E. antso*	Normal	10.78	2.0	Horn et al., 2014	Figure 2
sect. *Anthacanthae*	CROWN: *Athymalus*	Normal	24.56	5.0		Table 1
and sect. *Balsamis*	*Anthacanthae*	Normal	18.22	3.4		Table 1
	MRCA *Anthacanthae-Balsamis*	Normal	7.56	1.4		Figure 2
*E*. subg. *Esula*	MRCA *Aphyllis-Exiguae* II	Normal	10.36	2.3	Horn et al., 2014	Figure 2
sect. *Aphyllis*	CROWN: *Aphyllis*	Normal	7.37	2.0		Figure 2
*E.* subg. *Esula*	MRCA *Arvales-Esula*	Normal	10.98	2.4	Horn et al., 2014	Figure 2
sect. *Esula*	CROWN: *Esula*	Normal	8.6	2.4		Figure 2, node 5
(African clade)	*E. virgata* clade	Normal	5.4	1.4		Figure S2
*Camptoloma*	MRCA *Buddlejeae-Camptoloma*	Normal	20.0	6.0	Navarro-Pérez et al., 2013	Figure 2
	Buddlejeae	Normal	7.5	3.0		Figure 2
*Campylanthus*	MRCA *Digitalis-Plantago*	Lognormal (0.0)	38.0	0.2	Bell et al., 2010	Figure S11
	MRCA Plantago-Aragoa^*^	Lognormal (7.1)	1.5	1.0	Thiv et al., 2010	P. 610
*Cicer*	CROWN: *Cicer*	Normal	14.8	5.0	Lavin et al., 2005	Figure 3, node 80
*Colchicum*	MRCA *Gloriosa-Colchicum*	Normal	43.3	7.0	Chacón and Renner, 2014	Figure 3, node 128/Table 2
*Geranium* subg. *Robertium*	MRCA *Pelargonium-Geranium*	Normal	28.0	3.0	Fiz et al., 2008	Figure 3, node D
	CROWN: *Robertium*^§^	Lognormal (7.25)	1.0	1.0		P. 329
*Kleinia*	ROOT: Asteraceae^†^	Lognormal (47.5)	10.0	0.75	Barres et al., 2013	P. 872
	*Lordhowea insularis*	Lognormal (0.0)	7.0	1.0	Pelser et al., 2010	Table 1
*Plocama*	MRCA *Putorieae-Paederieae*	Normal	34.4	5.5	Bremer and Eriksson, 2009	Table 1

*At least one node (preferably toward the root) was constrained in each phylogeny (**Figures S1–S16** show resulting chronograms explicitly stating any constrained nodes)*.

* *Plantaginacearumpollis miocenicus (Late Miocene, 10.3 Ma; Nagy, 1963; Doláková et al., 2011)*.

§ *Geranium cf. lucidum (Late Miocene, 7.246 Ma ± 0.005; Van Campo, 1989)*.

† *Raiguenrayun cura (Middle Eocene, 47.5 Ma; Barreda et al., 2012)*.

### Diversification analyses

We used divergence times estimated above to calculate absolute diversification rates in the aforementioned lineages. There have been numerous developments in macroevolutionary birth-death models that allow a more accurate estimation of extinction and speciation rates from dated molecular phylogenies, including episodic time-variable models and trait-dependent diversification models (Stadler, 2013; Morlon, 2014; Rabosky et al., 2014). However, these methods usually require both very large phylogenies (e.g., ≥100 tips) and a fairly complete sampling. We here chose a simpler approach, the “method-of-moments” estimator (Magallón and Sanderson, 2001), implemented in the R package geiger (Harmon et al., 2008). This method uses clade size (extant species number) and clade age (either crown or stem) to estimate net diversification rates (*r* = speciation minus extinction), under different values of background extinction or turnover rate (ε = extinction/speciation = 0.0, 0.5, and 0.9). Net diversification rates (*bd.ms* function in geiger) were here estimated for all RF disjunctions and for a series of successively encompassing clades (e.g., section, genus, tribe, subfamily, and so on) to detect possible rate shifts. Crown diversification rates could not be estimated for clades containing only two taxa because Magallón and Sanderson's formula (*r* = [log(n)–log 2]/t in its simplest version, that is, with no extinction; for ε > 0 see formula number 7 in Magallón and Sanderson, 2001) results in zero in this case. In an attempt to counter this problem, clades containing two taxa were assigned a diversity value of 2.01, which permitted the estimation of net diversification rates (*r*).

Additionally, the probability of obtaining a clade with the same size and age as the RF disjunction, given the background diversification rate of the encompassing clade/s and at increasing extinction fractions (ε = 0, 0.5, and 0.9), was estimated with the *crown*.*p* function in geiger. We also estimated the 95% confidence interval of expected diversity through time (*crown.limits* function, geiger, ε = 0, 0.5, and 0.9) for a clade that diversifies with a rate equal to that of the family containing a RF disjunction with the highest diversification rate (i.e., Asteraceae); we then mapped RF lineages according to their crown or stem age and standing species diversity to assess which RF disjunct clades are significantly less diverse than expected given their stem and crown age in relation to the highest rate calculated for a RF family (Magallón and Sanderson, 2001; Warren and Hawkins, 2006).

## Results

### Divergence times

Up to 21 disjunctions were identified and divergence times were estimated for 17 lineages exhibiting a geographic distribution consistent with the RF pattern (Figures 3, 4 and Figures S1–S17). These disjunctions represent two possible geographic splits: I) Eastern Africa (including the Eastern Arc Mountains, the Horn of Africa, and Southern Arabia) vs. Southern Africa (including southern Angola and Namibia and the Cape Flora region up to the Drakensberg Mountains), hereafter E-S, and II) Western Africa (including Macaronesia and NW Africa south to the Cameroon volcanic line) vs. Eastern Africa, (with or without S Africa), hereafter W-E(&S).

**Figure 4:**
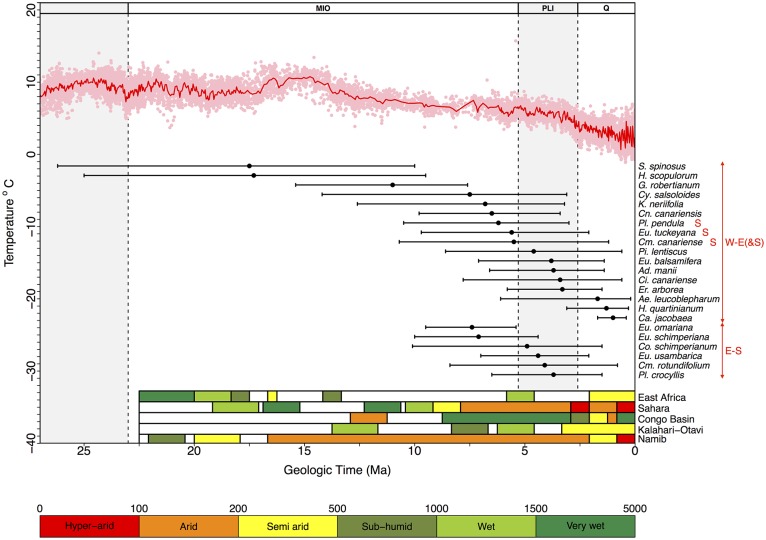
**Diagram showing estimated lineage divergence times (mean and 95% HPD confidence intervals) for Rand Flora disjunctions dated in this study and indicated in the MCC chronograms depicted in Figures S1–S17**. W-E(&S): divergence times estimated between disjunct taxa distributed in Macaronesia-NW-W Africa vs. Eastern Africa (a red S indicates presence in Southern Africa); E-S: estimated divergence times between disjunct taxa distributed in southern Arabia-Eastern Africa vs. southern Africa. The red line above represents the change in global temperatures over the Cenozoic as reflected by global-deep-sea oxygen records compiled from Zachos et al. (2008); colored bars in the right bottom corner indicate climatic conditions in five regions that underwent major climate changes—either desertification or tropicalization—during the Neogene (adapted from Senut et al., 2009). Taxa names correspond to those in Table 1, plus two groups from the literature: *Pistacia lentiscus* and *Erica arborea* (see Discussion).

From youngest to oldest, E-S disjunctions (Figure 4) occur in *Plocama* (ca. 4 Ma between S African *Pl. crocyllis* on one side and, among other E African-S Arabian species, *Pl. yemenensis* and *Pl. tinctoria* on the other; Figure 3 and Figure S15), *Camptoloma* (ca. 4 Ma between E African *Cm. lyperiiflorum* and S African *Cm. rotundifolium*; Figure 3 and Figure S4), *Colchicum* (ca. 5 Ma between E African *Co. schimperianum* and S African *Co. albanense* and *Co. longipes*, Figure 3 and Figure S8), the African clade of *Euphorbia* sect. *Esula* (ca. 7 Ma between S African and E African taxa; Figure 3 and Figure S10), and *E*. sect. *Anthacanthae* (ca. 7.5 Ma separate subsects. *Platycephalae* and *Florispinae*; Figure 3 and Figure S11).

Also from youngest to oldest, W-E disjunctions (Figure 4) can be found in the *Azorina* clade of *Campanula* (ca. 1 Ma between Cape Verdean *Ca. jacobaea* and Socotran *Ca. balfouri*; Figure 3 and Figure S3), in *Hypericum* sect. *Campylosporus* (ca. 1.5 Ma within *H. quartinianum*; Figure 3 and Figure S13), in *Aeonium* (1.7 Ma between E African *Ae. leucoblepharum* and a number of Macaronesian species; Figure 3 and Figure S2), in *Cicer* (ca. 3.5 Ma between Canarian *Ci. canariense* and E African *Ci. cuneatum*; Figure 3 and Figure S7), in *Adenocarpus* (ca. 4 Ma between E African *Ad. mannii* and a number of species in the *Ad. complicatus* complex; Figure 3 and Figure S1), in *Euphorbia* sect. *Balsamis* (ca. 4 Ma between W African *Eu. balsamifera* subsp. *balsamifera* and E African-S Arabian *Eu*. *balsamifera* subsp. *adenensis*; Figure 3 and Figure S11), in *Camptoloma* (ca. 5.5 Ma between Canarian *Cm. canariense*, on one hand, and E African *Cm. lyperiiflorum* and S African *Cm. rotundifolium*, on the other; Figure 3 and Figure S4), *Eu*. sect. *Aphyllis* (ca. 5.5 Ma between Cape Verdean *Eu. tuckeyana* and all E African and S African species in this section; Figure 3 and Figure S9), *Plocama* (ca. 6 Ma between Canarian *Pl. pendula* and S African *Pl. crocyllis* plus a number of E African/S Arabian *Plocama* species, Figure 3 and Figure S16), in *Canarina* (6.5 Ma between Canarian *Cn. canariensis* and E African *Cn. eminii*; Figure 3 and Figure S6), in *Kleinia* (ca. 7 Ma between the Macaronesian species, on one hand, and a clade of several E African species, on the other; Figure 3 and Figure S14), in *Campylanthus* (ca. 7.5 Ma between the Macaronesian and the E African-S Arabian species in the genus; Figure 3 and Figure S5), in *Geranium* subgen. *Robertium* (ca. 11 Ma between all E African species in this subgenus and a clade formed by W African taxa and a number of broadly distributed circum-Mediterranean and E Asian taxa; Figure 3 and Figure S12), in the *Androsaemum* clade of *Hypericum* (ca. 17 Ma between Socotran *H. scopulorum*, *H. tortuosum* and Turkish *H. pamphylicum*, on one hand, and a number of Macaronesian and W Mediterranean species, on the other; Figure 3 and Figure S13), and in *Sideroxylon* (ca. 17 Ma between Moroccan *S. spinosus* and E African *S. mascatense*; Figure 3 and Figure S16).

### Absolute diversification rates

**Figure 5:**
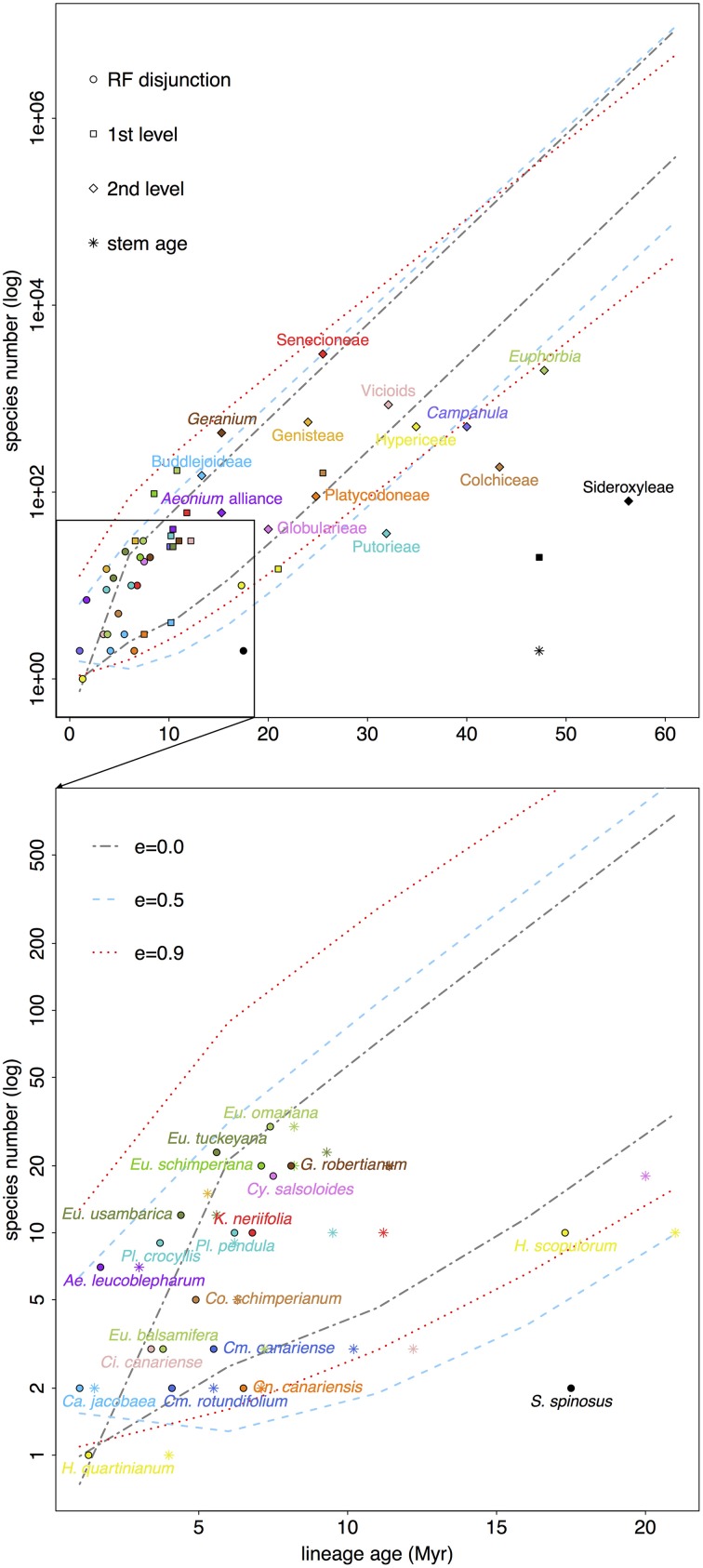
**RF lineages (names as in Table 1) are plotted according to their standing diversity (n) and age of the node (circle, crown; star, stem) corresponding to their disjunction (below)**. Successive encompassing lineages (above) also plotted (squares indicate the clade, section, subgenus the RF disjunct clade falls in; diamonds go one level above indicating genus, tribe, subfamily). Ninety five percent confidence intervals show expected diversity through time for a RF lineage that diversifies at the highest rate estimated (i.e., Asteraceae) given three possible scenarios: no extinction (ε = 0), turnover at equilibrium (ε = 0.5), and high extinction (ε = 0.9). See Table S1 for associated net diversification rate estimates.

Figure 5 and Table S1 show results from net diversification rate analyses. Most lineages fall within the 95% CI of expected diversity under a no-extinction scenario (ε = 0) in the context of the RF family showing the highest rate of diversification (i.e., Asteraceae). However, some RF disjunct clades were significantly less diverse: W-E disjunctions in *Sideroxylon* (*S. spinosus* vs. *S. mascatense*), *Canarina* (*C. canariensis* vs. *C. eminii*), and *Hypericum* (*H. canariense* clade vs. *H. scopulorum* and *H. pamphylicum*). Other RF disjunct taxa were above the upper bound of the 95% CI: W-E(&S) disjunction in *Euphorbia* sect. *Aphyllis* (S), *Adenocarpus, Aeonium*, and *Campanula*; and E-S disjunction in *Plocama*. Otherwise, all taxa fell within the 95% CI with increasing ε values 0.5 and 0.9, except for *Sideroxylon*.

Interestingly these trends are generally repeated in the more encompassing lineages of the least diverse RF disjunct clades (e.g., *Canarina, Hypericum*, *Sideroxylon*). Notably, though *Camptoloma* has a low extant diversity given its age (three species diverging in the last 6 Myr), the subfamily it belongs to, that is Buddlejoideae, stands above the 95% CI for ε = 0 (Figure 5). Something similar can be observed in the case of *Kleinia*, which shows lower diversity than its encompassing lineage, tribe Senecioneae. Another example of potential diversification shift, though in the opposite direction, is that of *Euphorbia*, where the genus is significantly less diverse than expected given its age (for all ε values) but RF disjunct clades are species-richer than expected (i.e., *E*. sect. *Aphyllis*), except for those that fall within the 95% CI limits (e.g., *E*. sect. *Balsamis*, Figure 5).

When comparing crown vs. stem age it is noticeable that in some RF disjunct clades crown and stem ages are far apart: *Cicer canariensis* vs. *Ci. cuneatum* (crown age = 3.4 Ma, stem age = 12.2 Ma, with the stem age falling below the lower bound of 95% CIs when ε = 0.0 and 0.9; Figure 5). Other examples include, *Camptoloma* (crown age= 5.5 Ma, stem age = 10.2 Ma), *Campylanthus* (crown age = 7.5 Ma, stem age = 20.0 Ma), and most notably *Sideroxylon* (crown age = 17.4 Ma, stem age = 47.3 Ma, Figure 5).

## Discussion

### Rand Flora disjunctions through time

Engler's (1910) intuition on the *Tertiary* origins of the Afro-Macaronesian floristic element, aka Christ's (1910) Rand Flora, very much hit the mark on the timing of its assembly. Our divergence estimates for Rand Flora disjunctions span five successive time frames (Figure 4): Burdigalian, Tortonian, and Messinian Stages (within the Miocene), the Pliocene, and the Pleistocene. The two earliest disjunctions happen on genera *Sideroxylon* and *Hypericum* and date back to the Early Miocene (Burdigalian; 17.5 and 17.3 Ma, respectively), coinciding with the longest warming period of the Miocene (the Miocene Climatic Optimum; Zachos et al., 2008) and with the start of desertification in south-central Africa (Senut et al., 2009). Couvreur et al. (2008) also dated divergences in Annonaceae back to this time period and explained them in terms of a once-continuous Early Miocene rainforest that became fragmented by decreasing moisture brought by the closure of the Tethys Sea. The fact that *Sideroxylon* and *Hypericum* exhibit less xeric affinities than other RF lineages, and that their crown diversification dates back to the Paleogene (Meseguer et al., 2013; Stride et al., 2014), suggests these taxa could be relicts of an earlier megathermal flora (sensu Morley, 2000, 2003).

The next disjunction is that of *Geranium* subgen. *Robertium* and it dates back to the Late Miocene (Tortonian, 11.0 Ma). This disjunction follows a drastic decline in global temperatures (Late Miocene cooling, 11.6–5.3 Ma; Beerling et al., 2012) and coincides with the temporary closing of the Panama isthmus in America and a moist “washhouse” climate period in Europe (Böhme et al., 2008). This disjunction marks the separation of Macaronesian (e.g., *G. maderense*) and circum-Mediterranean taxa (e.g., *G. robertianum*), on one side, and E African species (e.g., *G. mascatense*), on the other, leaving open the possibility of a colonization of Macaronesia by a Mediterranean ancestor (Figure 4 and Figure S12). Since the disjunction in *Geranium* subgen. *Robertium* is linked to a more humid period, rather than an increase on aridity, and because the possible Mediterranean origin of its Macaronesian taxa, this lineage does not exactly match the RF pattern.

Most other Neogene disjunctions seem to concentrate around the Miocene-Pliocene border (Figure 4). Messinian disjunctions can be observed in *Camptoloma, Campylanthus*, *Canarina*, *Euphorbia* sects. *Anthacanthae* and *Aphyllis*, *Kleinia*, and *Plocama*. Pliocene disjunctions are found in *Adenocarpus*, *Camptoloma*, *Cicer*, *Colchicum*, *Euphorbia*. sects. *Balsamis* and *Aphyllis*, and *Plocama*. These disjunctions follow two different geographic splits, W-E(&S) Africa and E-S Africa. W-E(&S) disjunctions present the widest temporal (as well as spatial) range. Besides the lineages dated here, other examples can be found in the literature of this W-E(&S) disjunction, e.g., according to Xie et al. (2014), in the Anacardiaceae *Pistacia lentiscus* and *P. aethiopica* diverged 4.55 Ma (see Figure S17). E-S disjunctions link South Africa and adjacent areas to the East African Rift Mountains, the Ethiopian Highlands, and the Arabian Peninsula. The timing of these E-S disjunctions (Mio-Pliocene) matches the uplift of the Eastern Arc Mountains (Sepulchre et al., 2006). The absence of W-S disjunctions is notable and probably results from African aridification having started in the early Miocene (some 17–16 Ma) in the region where the current Namib Desert stands. This aridification not only persisted through time in this area but also intensified and resulted in the formation of the Kalahari Desert (Senut et al., 2009), effectively limiting range expansions in this direction (W-S), in the absence of successful colonization following LDD. Even in the case of genus *Colchicum* (Figure S8), were S African species appear closely related to NW African ones, W Mediterranean species are always sister to E Mediterranean ones. These leaves open the possibility of a colonization of NW Africa (from S Africa) via E Africa and W Mediterranean populations with subsequent extinction in E Africa. An alternative colonization from Central-West Asia into South Africa and NW Africa seems unlikely given the phylogeny of this genus (Figure S8), though proper biogeographic inference to test either possibility remains to be done. Indeed, Sanmartín et al. (2010) found a higher frequency of biotic exchange between NW-E African elements than with either E-S African or W-S African ones, where the latter elements were hardly connected, if at all, confirming our observations. We further argue that the magnitude of observed biotic exchange follows the history of desertification in Africa.

In all, the sequential timing of Neogene disjunctions in RF lineages, which is nonetheless concentrated in certain time intervals (e.g., Late Miocene-Pliocene), is in agreement with a scenario of range expansions (dispersal) in favorable times (windows of opportunity) and range contractions (extinction) as aridification flared up. Extinction results in absence (of a population, species, clade, or lineage) and thus leaves hard to track traces in phylogenies in the absence of fossil data (Meseguer et al., 2015). If repeated cycles of speciation, dispersal, and extinction take place in the same area over time, only taxa that optimize any (or a combination) of these processes (e.g., increased speciation, higher dispersal, lower extinction rates) will persist. It is to be expected that more recent populations, species, clades, or lineages show traces of these processes when compared to ancient ones.

On the other hand, our net diversification rate estimates (Figure 5) do no fully support an extinction explanation since, in the context of the family with the highest diversification rate among RF lineages, i.e., Asteraceae, most of the taxa fall inside the 95% CI under a no-extinction scenario (ε = 0.0). However, the method chosen to estimate net diversification rates (Magallón and Sanderson, 2001), though more appropriate given phylogeny size and sampling effort, is still limited. Crown diversification rates cannot be estimated for clades with 2 terminal taxa (see Materials and Methods), which is the case for several RF lineages (e.g., *Sideroxylon*). Additionally, the “method-of-moments” estimator performs well detecting declining diversity for old groups in exceedingly species-poor clades (Magallón and Sanderson, 2001; Warren and Hawkins, 2006) or young groups notably species-rich (recent radiations, Magallón and Sanderson, 2001), but we observed that statistical power is low to detect declines in diversity for young species-poor groups (e.g., *Camptoloma*). Most RF disjunct clades dated comprise less than 10 species—e.g., *Aeonium*, *Campanula*, *Camptoloma*, *Cicer*, *Colchicum*, *Euphorbia* sect. *Balsamis*, *Kleinia*, and *Plocama*—, limiting our ability to effectively detect the effects of extinction.

Nonetheless, if we focus on crown ages, disjunct clades in *Canarina, Hypericum*, and *Sideroxylon* are less diverse than expected, and given that their encompassing lineages (Table 1, Figure 5) also follow this trend, it would be safe to assume these lineages have indeed experienced high levels of extinction through time. Likewise, if we were to focus on stem ages, a few other groups fall below the no-extinction scenario (ε = 0.0), notably, *Camptoloma*, *Campylanthus*, and *Cicer*. Moreover, these groups exhibit wide-spanning (often >10 Ma) stem-crown intervals (see *Sideroxylon* or *Cicer* in Figure 5), an observation that has been tied to historically high extinction rates in recent diversification studies (Antonelli and Sanmartín, 2011; Nagalingum et al., 2011). This would further support the hypothesis that lower diversification rates in RF lineages could be explained in terms of increased extinction rather than a decrease in speciation rates.

Additionally, and given the aforementioned limitations of our diversification method of choice, it would also be safe to conclude that, within *Euphorbia*, sects. *Anthacanthae* (sect. *Balsamis* included), sect. *Esula*, and sect. *Aphyllis*, present higher diversity than expected (above the CI for ε = 0.0 in all cases, and also above the CI for ε = 0.5 for the former two clades), which is exceptional in the context of the genus, since *Euphorbia* is significantly poorer than expected for all ε values. Horn et al. (2014) also detected increased diversification rates in these sections of *Euphorbia*. Desertification-tropicalization cycles in Africa (Senut et al., 2009) suggest repeated reconnections between now disjunct RF regions since the Neogene, which would have permitted biotic exchange in favorable periods, whereas the isolation of these regions at unfavorable times would have induced speciation through vicariance, enhancing endemicity in these sub-humid/sub-xeric lineages. Molecular dating in tropical trees from the genus *Acridocapus* (Malpighiaceae; Davis et al., 2002) and the Annonaceae family (Couvreur et al., 2008) shows a similar pattern of connection phases between East African and Guineo-Congolian rainforest regions since the Oligocene following major climate shifts.

The youngest disjunctions, those of *Aeonium*, *Campanula*, and *Hypericum* sect. *Campylosporus*, are Pleistocene in age (Figure 4) and far too recent to result from the Neogene aridification of the African continent. Either rare LDD (i.e., *Aeonium*; Kim et al., 2008) or stepping-stone dispersal events (i.e., *Campanula*, Alarcón et al., pers. comm.), perhaps favored by Pleistocene cool and drier glacial cycles, could explain these more recent disjunct geographic patterns, as previously observed in other African taxa, e.g., *Convolvulus* (Carine, 2005), *Moraea* (Galley et al., 2007), or the tree heath (*Erica arborea*). Désamoré et al. (2011) took notice of successive range expansions of *Er. arborea* from an Eastern African center of diversity toward Northwest Africa, Southwest Europe, and Macaronesia, first during the Late Pliocene (ca. 3 Ma; Figure 4) and subsequently in the Pleistocene (ca. 1 Ma).

### Redefining the Rand Flora pattern

In a recent review, Linder (2014) synthesized the individual histories of numerous African lineages by recognizing five different “floras,” which he defined as “groups of clades, which: (a) are largely found in the same area, (b) have largely the same extra-African geographical affinities, (c) share a diversification history, and (d) have a common maximum age.” The “Rand Flora” does not fit well this definition. This *flora* does group a number of lineages that share the same geographic range (even if discontinuous), but they have slightly different climatic tolerances, i.e., sub-humid to sub-xeric or xerophilic, and they do not necessarily share the same extra-African geographical affinities. Some RF lineages fall within what Linder (2014) terms “tropic-montane flora” (e.g., *Hypericum*, *Canarina*), others within the “arid flora” (e.g., *Kleinia*, *Campylanthus*). Some RF lineages are better connected with the Mediterranean Region (e.g., *Adenocarpus*), others with Asia and the Indo-Pacific Region (e.g., *Plocama*). Moreover, RF taxa on either side of any given disjunction (i.e., W-E or E-S) do no longer share a “diversification history,” though they do share the same fate as other RF lineages with similar distribution. In fact, the different ages estimated here for the various RF disjunctions agree well with what has been termed biogeographic *pseudocongruence* (Donoghue and Moore, 2003), a phenomenon whereby two or more lineages display the same biogeographic pattern but with different temporal origins (Sanmartín, 2014). What is shared by all RF lineages is the nature of the climatic (ecological) barriers separating the taxa at either side of any given disjunction: arid regions such as the Sahara, the Kalahari or the Namib deserts, or the tropical lowlands in Central Africa. The congruence between RF disjunction ages and successive major climatic events in Africa during the Neogene (Figure 4) suggest that the ongoing aridification of the continent (or the “tropicalization” of Central Africa) affected RF lineages according to their different physiological (climatic) tolerances: more sub-humid lineages diverged first (e.g., *Sideroxylon*), more xeric later (e.g., *Campylanthus*).

One point of contention in the literature has been the limits of the Rand Flora with respect to the “Arid Corridor” or “Arid Track” (hereafter AC), a path repeatedly connecting south-west to north-east arid regions in Africa (and henceforth to central and southwest Asia) first proposed by Winterbottom (1967) and later expanded by de Winter (1966, 1971) and Verdcourt (1969). Bellstedt et al. (2012) defined the AC pattern as the disjunction occurring between Southern Africa and Eastern African-Southern Arabian xeric floristic elements. Linder (2014) considered the RF as an expansion of the AC to the west, in agreement with Jürgens' (1997) view. However, we consider that the RF and AC patterns are different. AC elements have more xeric preferences than the sub-humid to sub-xeric ones exhibited by RF elements. AC elements often extend into deserts (e.g., Namib, Kalahari, Sahara)—see studies by Beier et al. (2004) on *Fagonia* (Zygophyllaceae), Bellstedt et al. (2012) on *Zygophyllum* (also Zygophyllaceae), Carlson et al. (2012) on *Scabiosa* (Dipsacaceae), or Bruyns et al. (2014) on Ceropegieae— and have broader, more continuous distributions, plus they tend to be younger in age (often Pleistocene, coincident with Quaternary glaciation cycles). Our understanding is that this younger xeric AC elements move in parallel to RF taxa webbing with them in areas favorable to either, and thus confusing their limits. Something similar could have happened with Afromontane elements migrating south to north as the Eastern African mountains rose through the Mio-Pliocene; these elements are not part of the RF (e.g., *Iris*, *Moraea*, Galley et al., 2007).

In this study, we have provided a temporal framework for the Rand Flora pattern and estimated net diversification rates for 17 RF lineages. Our results provide some support to the historical view of an ancient African flora, whose current disjunct distribution was probably modeled by the successive waves of aridification events that have affected the African continent starting in the Miocene, but whose origin predates the latest events of Pleistocene climate change. These patterns were probably formed by a combination of climate-driven extinction and vicariance within a formerly widespread distribution. Whether these lineages all had a continuous, never interrupted, distribution that occupied all the area that now lies in between the extremes of the disjunction, or they had a somewhat narrower distribution in the past and they expanded their range tracking their habitat across the landscape in response to changing climate (e.g., along a corridor), is difficult to say with the current evidence. Discerning between these hypotheses will require the integration of phylogenetic, biogeographic and ecological approaches to reconstruct the ancestral ranges and climatic preferences of ancestral lineages (Mairal et al., 2015; Meseguer et al., 2015). Compared to speciation, extinction has received far less attention in studies focusing on the assembly of tropical biotas. Disentangling extinction from other processes is particularly difficult because the biodiversity we observe today is only a small fraction of that of the past. The Rand Flora pattern might offer a prime study model to understand the effects of climate-driven extinction in the shaping of continent-wide biodiversity patterns.

## Author contributions

IS and LP conceived and designed the study. LP analyzed the data with help from IS, RR, and MM. LP and IS co-wrote the text, with contributions from MH, RR, MM, and AM. All authors contributed with data compilation, figure preparation, or text comments. MM has copyright of all plant pictures, except for *Cicer canariense*.

### Conflict of interest statement

The authors declare that the research was conducted in the absence of any commercial or financial relationships that could be construed as a potential conflict of interest.

## Supplementary material

Supplementary Materials include descriptions of study groups with references, Table S1, and Figures S1–S17.

Table S1 **Net diversification rates (bd.ms) for all RF disjunct clades and their encompassing lineages (bold = highest crown.p, red when *n* ≤ 2) under three possible scenarios: no extinction (ε = 0), turnover at equilibrium (ε = 0.5), and high extinction (ε = 0.9)**. Probability (crown.p) of obtaining a clade with the same size and age as the RF disjunction, given the background diversification rate of the encompassing clade/s and at increasing extinction fractions (bold = highest crown.p, italics *p* < 0.05). Stem and Crown ages in Myr.

Figures S1–S17 **BEAST MCC chronograms showing mean estimates and 95% high posterior density (HPD) confidence intervals for those nodes receiving 50% support**. Branch width is proportional to PP support. Red colored taxa indicate Eastern African provenance; Macaronesia/western African taxa and southern African taxa are colored in blue and green, respectively. Calibration points are indicated with stars; RF disjunctions within each lineage discussed in the text and represented in Figures 3–5 are indicated with arrows.
