# Supplementary material for: Living on the edge: timing of Rand Flora disjunctions congruent with ongoing aridification in Africa
Source: Front Genet. 2015 May 1;6:154. doi: 10.3389/fgene.2015.00154 (PMC4416453; doi:10.3389/fgene.2015.00154)
Supplement: Table S1 — Net diversification rates (bd.ms) for all RF disjunct clades and their encompassing lineages (bold = highest crown.p, red when n ≤ 2) under three possible scenarios: no extinction (ε = 0), turnover at equilibrium (ε = 0.5), and high extinction (ε = 0.9). Probability (crown.p) of obtaining a clade with the same size and age as the RF disjunction, given the background diversification rate of the encompassing clade/s and at increasing extinction fractions (bold = highest crown.p, italics p < 0.05). Stem and Crown ages in Myr. [file DataSheet1.PDF]

STUDY GROUPS

1) *Adenocarpus* DC. (Fabaceae) (Fig. 3) contains approximately 20 species, and most of these inhabit NW Africa and the Iberian Peninsula. However, one well-supported monophyletic group (Percy and Cronk, 2002) shows a disjunct pattern where the three Macaronesian species and the widespread Mediterranean *A. complicatus* J. Gay form a group sister to the only subtropical species in Africa, *A. mannii* (Hook. f.) Hook. f. According to Percy and Cronk (2002) the distribution of *A. mannii* can be explained by either a W-E dispersal route of Mediterranean-Macaronesian species via the Saharan mountains or an eastern route of dispersal of Mediterranean species via the Red Sea Hills. Despite efforts to reconstruct its biogeographic history, Cubas et al. (2010) could not distinguish between these to routes in part due to a lack of support in the node leading to this species.

2) Aeonium alliance (Crassulaceae): although the Crassulaceae are a morphologically diverse, large family (ca. 1500 spp.), the Macaronesian representatives constitute a well-supported monophyletic lineage (Mort et al., 2002), which includes three genera endemic to Macaronesia —*Aichryson* Webb & Berthel. (10 spp.), *Greenovia* Webb & Berthel. (4 spp.), and *Monanthes* Haw. (10 spp.)— and genus *Aeonium* Webb & Berthel. (40 spp.), which comprises 37 Macaronesian endemic species, one species endemic to Morocco and two E African endemics (*A. leucoblepharum* Webb ex A. Richard and *A. stuessyi* H.Y. Liu). The molecular phylogeny reconstructed by Mort et al. (2002) revealed that *Aeonium* is paraphyletic, with *Greenovia* embedded within. The Moroccan species (*A. korneliuslemsii* H.Y. Liu) and the eastern African *A. leucoblepharum* appear nested within the Canarian radiation clade, and are related, although weakly supported, to the Cape Verde endemism *A. gorgoneum* J. A. Schmidt (Mort et al., 2007; Kim et al., 2008).

3) *Campanula* L. s.s., *Azorina* group (Campanulaceae): this clade of ca. 30 species (Mansion et al., 2012) is mainly distributed in North Africa but also includes

species from the Iberian Peninsula, South Arabia, Socotra, Eastern Africa, West Asia, and Macaronesian endemics. Alarcon et al. (2013) showed that *C. jacobaea* C. Sm. ex Webb (native to the Cape Verde archipelago) and *C. balfourii* Wagn. & Vierh. (endemic to Socotra) are sister species, with northwestern African endemic *C. hypocrateriformis* Dobignard as their closest known relative.

4) Camptoloma Benth. (Scrophulariaceae) is a small genus (3 spp.) with a widely disjunct African distribution: *Camptoloma canariense* (Webb & Berthel.) Hilliard is endemic to Gran Canaria, whereas *C. lyperiiflorum* Hilliard is found in the Horn of Africa, and *C. rotundifolium* Benth. is restricted to southern Africa (i.e., Angola, and South Africa). Broad-scale phylogenetic analyses within the family (Kornhall et al., 2001; Oxelman et al., 2005) have revealed that the genus is phylogenetically and geographically positioned between the Holarctic tribe Schrophularieae and the Southern Hemisphere tribes Manuleeae and Seleagineae (see also Hilliard, 1999). South African *Phygelius capensis* E. Mey. ex Benth., is reconstructed as sister to Camptoloma, though with low support, both of them are related to tribes Teedieae (4 genera, ca. 10 spp.) and Buddlejeae (4 genera, ca. 100 spp.). *Camptoloma* is monophyletic, with South African species *C. rotundifolium* sister to a clade comprising *C. canariense* and *C. lyperiiflorum* (Kornhall et al., 2001; Oxelman et al., 2005). This wide E-W disjunction and restricted species-level distribution make Camptoloma an interesting candidate to study the effect of contraction-extinction (aridification) events across the Sahara.

5) *Campylanthus* Roth. (Plantaginaceae): this is a small paelotropical genus (ca. 20 species) of shrubs and subshrubs with a markedly disjunct distribution. Two species are endemic to Macaronesia, *C. salsoloides* (L. f.) Roth from the Canary Islands, and *C. glaber* Benth. from Cape Verde, while the remaining species occur in the Horn of Africa, East Africa, Southern Arabia and Pakistan (Hjertson et al., 2008). A

new molecular phylogeny, including 14 species, confirmed the disjunction between the Macaronesian endemics and the East Africa-South Arabian clade (Thiv et al., 2010).

6) *Canarina* L. belongs to tribe Platycodoneae (ca. 90 spp.), an early diverging lineage within family Campanulaceae with nine genera, all except Canarina confined to East and Central Asia. The genus comprises only three species showing an extreme disjunction pattern. *Canarina eminii* Aschers. ex Schweinf., and *C. abyssinica* Engl. occur in East Africa, appearing mainly in "sky islands", but they differ in habitat and life-forms. *Canarina eminii* is an epiphytic herb endemic to the highly fragmented Afromontane forest belts, extending from the Ethiopian Highlands to Malawi. *Canarina abyssinica* is a rock dweller of upland forests, restricted to few patches in East Africa, from Southern Sudan to Tanzania. On the other side of the distribution appears *C. canariensis* (L.) Vatke., which is restricted to the edges of the laurisilva forest in the Canary Islands. The Asian genus *Ostrowskia* is the closest relative of genus *Canarina* (Mansion et al., 2012).

7) Cicer L. is a Fabaceae genus that belongs in subfamily Papilionoideae, usually with herbaceous or semi-shrubby biotypes. The genus seems to have originated in the Early Miocene Neotethys, and consists of ca. 40 species distributed throughout the Northern Hemisphere (Javadi et al., 2007). The Canarian endemic *C. canariense* A.Santos & G.P.Lewis and the Eastern African *C. cuneatum* A.Rich. (Egypt, Ethiopia, Sudan, and Saudi Arabia) form an early-diverging strongly supported clade sister to the remaining species of *Cicer*, though probably Moroccan endemic *C. atlanticum* Coss. Ex Maire (see monograph by van der Maesen, 1972), which was not sampled for DNA, also falls in this clade. Javadi et al. (2007) suggest that the geographical disjunction present in these species could be explained in terms of their ancestor having had a broader distribution in more favorable climate. They are indeed the only ones with climbing habits found in relatively humid habitats.

8) Colchicum L. (Colchicaceae): former genus *Androcymbium* Willd. is paraphyletic in relation to *Colchicum* (Vinnersten & Reeves 2003, Manning et al. 2007) and has therefore been transferred to *Colchicum* (Manning et al. 2007) together with *Bulbocodium* L. and *Merendera* Ramond. While *Colchicum* s. str., together with *Bulbocodium* and *Merendera*, are genera centered in the Mediterranean Basin and eastwards, the ca. 60 species formerly belonging to *Androcymbium* are mainly found in Africa, with several disjunctions between south, east, and northwest, and a high concentration of species (ca. 50) in southern Africa, especially in the winter-rainfall region. The disjunct distribution between south and north Africa is according to Caujapé-Castells et al. (2001) best explained by an origin in southern Africa, followed by dispersal northwards via the African "Arid Corridor", prior to the desiccation of Africa in the Miocene.

9) *Euphorbia* subgen. *Athymalus* (sects. *Anthacanthae* and *Balsamis*) (Euphorbiaceae): This subgenus —with about 150 species and a great diversity of life forms including herbs, geophyte, shrubs, small trees and numerous succulent forms— has its main center of diversity in southern Africa and a secondary center in the Horn of Africa. Most species in this group have restricted geographic ranges. The exception is *E. balsamifera* Aiton (sect. *Balsamis*), which has a wide distribution exhibiting the W-E Rand Flora pattern. *Euphorbia balsamifera* subsp. *balsamifera* is distributed in the Canary Islands and the west coast of Africa (Morocco and Mauritania), whereas *E. balsamifera* subsp. *adenensis* (Deflers) Bally occurs in eastern Africa (i.e., Ethiopia, Somalia, NE Sudan), the southern Arabian Peninsula (Yemen, Oman) and in one locality in Socotra. A third morphologically divergent (long linear leaves) subspecies, *E. balsamifera* subsp. *sepium* (N.E.Br.) Maire, occurs in the western Sahel region. Section *Balsamis* also includes taxa from the southern Arabian Peninsula (Peirson et al., 2013). Section *Anthacanthae* is composed of mainly southern African succulent species, though subsect. *Platycephalae* (e.g., *E. grantii* Oliv., *E. platycephala* Pax, *E.*

omariana M.G. Gilbert), which is sister to subsect. *Florispinae*, has a wider distribution throughout eastern Africa.

10) *Euphorbia* L. subgen. *Esula* sect. *Aphyllis* (Euphorbiaceae): This section represents one of the few introductions of the mostly northern temperate subgenus *Esula* into non-Mediterranean Africa. Section *Aphyllis* consists of two subclades — subsect. *Africanae* and subsect. *Macaronesicae*— whose names reflect their disjunct distribution (Barres et al., 2011; Riina et al., 2013). Subsection *Macaronesicae* is found in Macaronesian islands (except for the Azores) and the Atlantic coast of both Morocco and Western Sahara, from sea level to 1600(–2200) m. Species in subsect. *Africanae* occur in continental Africa (mainly central-eastern and southern Africa), southern Arabian Peninsula, Socotra, and Madagascar, from 100 to 2300 m. Members of sect. *Aphyllis* are shrubs, succulent to semi-succulent or sub-woody, much-branched or dendroid, some with branches pencil-like, often green (chlorophyllous). They grow in arid to semi-arid habitats such as stony plains and slopes, sparsely woody grasslands, loamy to sandy areas, often associated with low and open vegetation in which succulents predominate. A key physiological feature present in these species, as well as in other subclades within Euphorbia, is the crassulacean acid metabolism (CAM), a common physiological adaptation in plants growing in water limited habitats (Horn et al., 2014).

11) *Euphorbia* subgen. *Esula* sect. *Esula* (African clade): Another lineage within subgen. *Esula* present in Africa, and interesting because of some of its members exhibit a S-E Rand Flora disjunction, is a small subclade (here informally named "AfrEsula" clade) of ca. 10 species within sect. *Esula* (Riina et al., 2013). Most of the species in the AfrEsula clade are ericoid-leaved perennial herbs and shrubs (e.g. *E. ericoides* Lam., *E. genistoides* P.J. Bergius) distributed in southern and central-eastern Africa, Madagascar and Réunion, with one species reaching the Arabian Peninsula. The southern African species occur in the mesic parts of the winter-rainfall Cape

floristic region, in the summer-rainfall grasslands and forest margins of Natal, and extending into Zimbabwe and Mozambique. One species, *E. cyparissioides,* shows the broadest distribution range, occurring from Nigeria to east and south tropical Africa.

12) *Geranium* subgen. *Robertium* (Picard) Rouy (Geraniaceae): genus *Geranium* L. is divided into two subgenera (Fiz et al., 2008): *Geranium* (>380 species) and *Robertium* (24 species). Within the subgenus *Robertium*, the species endemic to Macaronesia, Morocco and the Iberian Península form the sister group to a clade formed by species from E Africa. Together these two clades are sister to a clade of Asian species. Fiz et al. (2008) suggested that the Afro-Macaronesian clade originated from Asian lineages that migrated across the Arabian Plate, after its collision with Eurasia in the Miocene.

13) *Hypericum* L. (Hypericaceae): This large genus comprises ca. 500 species distributed in most temperate regions around the world, but it is also found in tropical and subtropical mountains. In Africa there are ca. 50 species distributed in Macaronesia, NW Africa, Eastern African Mountains and S Africa. A Rand Flora disjunction based on morphological similarities has been described between the Macaronesian section (i.e., *Webbia*) and the African section *Campylosporus* (Robson, 1985). A large-scale analysis, covering nearly 250 species worldwide and nuclear and plastid markers, shows that African species do not form a monophyletic group, but appear scattered along the phylogeny in at least five different clades, indicating independent biogeographic histories or, alternatively, an ancestral presence in Africa decimated by extinction (Meseguer et al., 2013). In some clades, a disjunction can be observed between Macaronesia-NW Africa and East Africa, such as *H. somaliense* N. Robson (Somalia)-*H. tomentosum* Durand ex Steud. (Mediterranean) or *H. canariense* L. (Canary Islands)-*H. socotranum* R.D. Good (Socotra). An interesting disjunction at the population level is present in the Afromontane, closely related species *H. bequaertii* De Wild., *H. revolutum* Vahl, *H. roeperianum* G.W. Schimp. ex A. Rich., *and H.*

quartinianum A. Rich., with isolated populations at both sides of Africa: Eastern African mountains and the Cameroon volcanic line.

14) *Kleinia* Mill. (Asteraceae)*:* This gynuroid genus nested in *Senecio* L. s. l. comprises ca. 50 species, distributed mainly in E Africa, S Africa, and Madagascar, but also present in N Africa, with one species in the Canary Islands, 2 in northwest Africa (Maghreb), and 5–6 species in S Arabia and Socotra; in addition, 2–5 species are found in India and Sri Lanka. The taxonomic delimitation of *Kleinia* with genus *Senecio* has hindered the systematics of the former. A recent molecular phylogeny of Senecioneae (Pelser et al., 2007), based on nuclear ITS, showed that *Kleinia* forms a well-supported monophyletic group. Two sister lineages were recognized: one formed by Arabian, E African and S African species, and another comprising sub-Saharan and NW African species. Within the latter group, there is a clade with a W-E geographic disjunction: Canarian and NW African species —*K. neriifolia* Haw. and *K. anteuphorbium* (L.) Haw.— form a group which is sister to a clade that includes species from Southern Arabia, East Africa and Madagascar.

15) *Plocama* W. Aiton is a genus in the Rubiaceae included in tribe Putorieae with ca. 30 species, which generally grow as shrubs characterized by its fleshy indehiscent fruits. Backlund et al. (2007) reviewed tribe Putorieae identified several well-supported clades when reconstructing the phylogeny, but failed to identify morphological synapomorphies, which lead to the synonimization of a number of genera within Putorieae (e.g. Gaillonia A. Rich. ex DC., Crocyllis E. Mey. ex Benth., etc.). One of these clades consists of a number of disjunct species native to W, E and S Africa. *Plocama tinctoria* (Balf. f.) M. Backlund & Thulin (from Socotra and Somalia) and *P. yemenensis* (Thulin) M. Backlund & Thulin (from Oman and continental Yemen) form a clade sister to *P. crocyllis* (Sond.) M. Backlund & Thulin (from Namibia and South Africa). Meanwhile *P. pendula* Aiton (Canary Islands) is sister to the aforementioned species. Though Socotran *P. puberula, P. putorioides*, and *P.*

thymoides, Somali *P. calcicola* and *P. somaliensis*, and Yemeni *P. jolana* were not sample for DNA, they probably belong in the aforementioned E African-S Arabian clade (Backlund et al., 2007).

16) *Sideroxylon* L. (Sapotaceae)*:* Tribe Sideroxylae comprises ca. 80 species distributed among three genera, namely *Sideroxylon* (ca. 75 spp.), *Nesoluma* Baill. (three spp.), and *Argania* Roem. & Schult. (one sp.). Smedmark et al. (2006) showed that both *Nesoluma* and *Argania* were nested within larger *Sideroxylon*. Two sister species within this tribe are of interest to us: Moroccan *Argania spinosa* (L.) Skeels (originally described as Sideroxylon spinosum) and E African/S Arabian *Sideroxylon mascatense* (A. DC.) T.D. Penn. (Smedmark et al., 2006; Smedmark and Anderberg, 2007).

REFERENCES

Alarcón, M., Roquet, C., García-Fernández, A., Vargas, P., and Aldasoro, J. J. (2013). Phylogenetic and phylogeographic evidence for a Pleistocene disjunction between *Campanula jacobaea* (Cape Verde Islands) and *C. balfourii* (Socotra). *Mol. Phylogenet. Evol.* 69(3), 828–836.

Backlund, M., Bremer, B., and Thulin, M. (2007). Paraphyly of Paederieae, recognition of Putorieae and expansion of Plocama (Rubiaceae-Rubioideae). *Taxon* 56(2), 315–328.

Barres, L., Vilatersana, R., Molero, J., Susanna, A., and Galbany-Casals, M. (2011). Molecular phylogeny of *Euphorbia* subg. *Esula* sect. *Aphyllis* (Euphorbiaceae) inferred from nrDNA and cpDNA markers with biogeographic insights. *Taxon* 60(3), 705–720.

Caujapé-Castells, J., Jansen, R. K., Membrives, N., Pedrola-Monfort, J., Montserrat, J. M., and Ardanuy, A. (2001). Historical biogeography of *Androcymbium* willd. (Colchicaceae) in Africa: evidence from cpDNA RFLPs. *Bot. J. Linn. Soc.* 136(4), 379–392.

Cubas, P., Pardo, C., Tahiri, H., and Castroviejo, S. (2010). Phylogeny and evolutionary diversification of *Adenocarpus* DC. (Leguminosae). *Taxon* 59(3), 720–732.

Fiz, O., Vargas, P., Alarcón, M., Aedo, C., García, J. L., & Aldasoro, J. J. (2008). Phylogeny and historical biogeography of Geraniaceae in relation to climate changes and pollination ecology. *Syst. Bot.* 33(2), 326–342.

Hilliard, O. M. (1999). *The tribe Selagineae (Scrophulariaceae)*. Kew: Royal Botanic Gardens.

Hjertson, M., Henrot, J., and Thulin, M. (2008). *Campylanthus hajarensis* sp. nov. and a new record of *Campylanthus* (Scrophulariaceae) from Oman. *Nord. J. Bot.* 26(1–2), 35–37.

Horn, J. W., Xi, Z., Riina, R., Peirson, J. A., Yang, Y., Dorsey, B. L., Berry, P. E., Davis C. C., and Wurdack, K. J. (2014). Evolutionary burst in *Euphorbia* (Euphorbiaceae) are linked with Photosynthetic pathway. *Evolution*, 68(12), 3485–3504.

Javadi, F., Wojciechowski, M. F., and Yamaguchi, H. (2007). Geographical diversification of the genus *Cicer* (Leguminosae: Papilionoideae) inferred from molecular phylogenetic analyses of chloroplast and nuclear DNA sequences. *Bot. J. Linn. Soc.* 154(2), 175–186.

Kim, S. C., McGowen, M. R., Lubinsky, P., Barber, J. C., Mort, M. E., & Santos-Guerra, A. (2008). Timing and tempo of early and successive adaptive radiations in Macaronesia. *PLoS ONE* 3(5), e2139.

Kornhall, P., Heidari, N., and Bremer, B. (2001). Selagineae and Manuleeae, two tribes or one? Phylogenetic studies in the Scrophulariaceae. *Plant Syst. Evol.* 228(3–4), 199–218.

Manning, J., Forest, F., and Vinnersten, A. (2007). The genus *Colchicum* L. redefined to include *Androcymbium* Willd. based on molecular evidence. *Taxon* 56(3), 872-882.

Mansion, G., Parolly, G., Crowl, A. A., Mavrodiev, E., Cellinese, N., Oganesian, M., Fraunhofer, K., Kamari, G., Phitos, D., Haberle, R., Akaydin, G., Ikinci, N., Raus, T., and Borsch, T. (2012). How to handle speciose clades? Mass taxon-sampling as a strategy towards illuminating the natural history of *Campanula* (Campanuloideae). *PloS ONE* 7(11), e50076.

Meseguer, A. S., Aldasoro, J. J., and Sanmartín, I. (2013). Bayesian inference of phylogeny, morphology and range evolution reveals a complex evolutionary history in St. John's wort (*Hypericum*). *Mol. Phylogenet. Evol.* 67(2), 379–403.

Mort, M. E., Soltis, D. E., Soltis, P. S., Francisco-Ortega, J., and Santos-Guerra, A. (2002). Phylogenetics and evolution of the Macaronesian clade of Crassulaceae inferred from nuclear and chloroplast sequence data. *Syst. Bot.* 27(2), 271–288.

Mort, M. E., Soltis, D. E., Soltis, P. S., Santos-Guerra, A., and Francisco-Ortega, J. (2007). Physiological evolution and association between physiology and growth form in *Aeonium* (Crassulaceae). *Taxon*, 453–464.

Oxelman, B., Kornhall, P., Olmstead, R. G., and Bremer, B. (2005). Further disintegration of Scrophulariaceae. *Taxon* 54(2), 411–425.

Peirson, J. A., Bruyns, P. V., Riina, R., Morawetz, J. J., and Berry, P. E. (2013). A molecular phylogeny and classification of the largely succulent and mainly African *Euphorbia* subg. *Athymalus* (Euphorbiaceae). *Taxon* 62(6), 1178–1199.

Pelser, P. B., Nordenstam, B., Kadereit, J. W., and Watson, L. E. (2007). An ITS phylogeny of tribe Senecioneae (Asteraceae) and a new delimitation of Senecio L. *Taxon 56*(4), 1077–1077.

Percy, D. M., and Cronk, Q. C. (2002). Different fates of island brooms: contrasting evolution in *Adenocarpus*, *Genista*, and *Teline* (Genisteae, Fabaceae) in the Canary Islands and Madeira. *Am. J. Bot.* 89(5), 854–864.

Riina, R., Peirson, J. A., Geltman, D. V., Molero, J., Frajman, B., Pahlevani, A., Barres, L., Morawetz, J. J., Salmaki, Y., Zarre, S., Kryukov, A., Bruyns, P. V., and Berry,

P. E. (2013). A worldwide molecular phylogeny and classification of the leafy spurges, *Euphorbia* subgenus *Esula* (Euphorbiaceae). *Taxon* 62(2), 316–342.

Robson, N.K.B. 1985. Studies in the genus *Hypericum* L. (Guttiferae). 3. Sections 1. *Campylosporus* to 6a. *Umbraculoides*. *Bull. Br. Mus. (Nat. Hist.) Bot. Ser.* 12, 163–211.

Smedmark, J. E., Swenson, U., and Anderberg, A. A. (2006). Accounting for variation of substitution rates through time in Bayesian phylogeny reconstruction of Sapotoideae (Sapotaceae). *Mol. Phylogenet. Evol.* 39(3), 706–721.

Smedmark, J. E., and Anderberg, A. A. (2007). Boreotropical migration explains hybridization between geographically distant lineages in the pantropical clade Sideroxyleae (Sapotaceae). *Am. J. Bot.* 94(9), 1491–1505.

Thiv, M., Thulin, M., Hjertson, M., Kropf, M., and Linder, H. P. (2010). Evidence for a vicariant origin of Macaronesian–Eritreo/Arabian disjunctions in *Campylanthus* Roth (Plantaginaceae). *Mol. Phylogenet. Evol.* 54(2), 607–616.

van der Maesen, L. J. G. (1972). *Cicer* L., a monograph of the genus, with special reference to the chickpea (*Cicer arietinum* L.), its ecology and cultivation. Wageningen: Veenman H. and Zonen N.V., 341 p.

Vinnersten, A., and Reeves, G. (2003). Phylogenetic relationships within Colchicaceae. *Am. J. Bot.* 90(10), 1455–1462.

Table S1. Net diversification rates (bd.ms) for all RF disjunct clades and their encompassing lineages (bold = highest crown.p, red when n≤2) under three possible scenarios: no extinction (ε = 0), turnover at equilibrium (ε = 0.5), and high extinction (ε = 0.9). Probability (crown.p) of obtaining a clade with the same size and age as the RF disjunction, given the background diversification rate of the encompassing clade/s and at increasing extinction fractions (bold = highest crown.p, italics p<0.05). Stem and Crown ages in Myr.

				**bd.ms**			**crown.p**		
**Lineage**	**Richness**	**Stem**	**Crown**	**0.0**	**0.5**	**0.9**	**0.0**	**0.5**	**0.9**
Ad. mannii	15	5.3	3.7	**0.54456840**	0.48213740	0.22226870	**0.42537290**	0.39819950	0.38212080
Adenocarpus	30	9.4	6.6	0.41031060	0.37111330	**0.19830660**	0.15449390	0.21772780	**0.32660400**
Genisteae	560	41.2	24.0	0.23478290	0.22286220	**0.16624820**	*0.00548584*	*0.03543695*	**0.24812990**
Fabaceae	18860	71.0	61.0	0.15002710	0.14531170	**0.12280980**	*0.00012871*	*0.00371182*	**0.14011130**
Angiosperms	262196	–	132.0	0.08927046	0.08709107	**0.07668942**	*0.00000071*	*0.00010133*	***0.04302373***
Ae. leucoblepharum	**7**	3.0	1.7	**0.73691940**	0.63558720	0.24384520	**0.45155500**	0.42202850	0.40046890
Aeonium	40	14.2	10.4	0.28805120	0.26249090	**0.14781760**	*0.03536212*	0.09026290	**0.23454930**
Aeonium alliance	60	18.8	15.3	0.22230050	0.20445530	**0.12286310**	*0.01366463*	*0.04853158*	**0.18531150**
Crassulaceae	1500	56.0	43.0	0.15395520	0.14727870	**0.11547230**	*0.00313954*	*0.01889902*	**0.17038110**
Angiosperms	262196	–	132.0	0.08927046	0.08709107	**0.07668942**	*0.00029312*	*0.00315710*	**0.09121793**
Ca. jacobea	**2.01**	1.5	1.0	**0.00498754**	0.00496327	0.00386251	**0.95778500**	0.95494310	0.91589870
Azorina clade	26	11.2	10.1	**0.25395540**	0.22877140	0.11885870	**0.99280450**	0.80424570	0.70180750
Campanula	500	42.5	40.0	**0.13803650**	0.13088890	0.09696299	**0.98825780**	0.83944730	0.68768560
Campanulaceae	2400	80.0	76.0	**0.09329048**	0.08951007	0.07148787	**0.98499850**	0.86787070	0.67857540
Angiosperms	262196	–	132.0	**0.08927046**	0.08709107	0.07668942	**0.98462000**	0.86987020	0.67932940
Cm. rotundifolium	**2.01**	5.5	4.1	**0.00121647**	0.00121055	0.00094207	**0.95778500**	0.95494310	0.91589870
Cm. canariense	**3**	10.2	5.5	**0.07372093**	0.06395225	0.02141510	**0.55555560**	0.52082350	0.47325100
Camptoloma + *Phygelius*	**4**	10.6	10.2	**0.06795561**	0.05811604	0.01985904	**0.52645660**	0.50135390	0.46083480
Buddlejoideae	150	25.5	13.3	**0.32462320**	0.30343650	0.20412470	**0.97186820**	0.87383000	0.91676350

				bd.ms			crown.p		
**Lineage**	**Richness**	**Stem**	**Crown**	**0.0**	**0.5**	**0.9**	**0.0**	**0.5**	**0.9**
Scrophulariaceae	4800	43.0	38.5	**0.20216170**	0.19469420	0.15907430	**0.89180050**	0.77090530	0.88183380
Angiosperms	262196	–	132.0	0.08927046	0.08709107	**0.07668942**	0.62542940	0.58489290	**0.74284480**
Cy. salsoloides	18	13.7	5.1	**0.43082830**	0.38375150	0.18441020	**0.42194630**	0.39510270	0.37969900
Campylanthus	20	20.0	7.5	0.30701130	0.27438570	**0.13494740**	0.10214550	0.16017020	**0.22844080**
Globularieae	40	18.8	13.7	0.21866660	0.19926320	**0.11221190**	*0.01084014*	*0.04471008*	**0.15760690**
Plantaginaceae	1600	61.0	42.0	0.15915740	0.15232110	**0.11974940**	*0.00067409*	*0.01078351*	**0.18079200**
Angiosperms	262196	–	132.0	0.08927046	0.08709107	**0.07668942**	*0.00000114*	*0.00018943*	**0.06041009**
**Cn. canariensis**	**2.01**	8.2	6.5	**0.00076731**	0.00076358	0.00059423	**0.95778500**	0.95494310	0.91589870
Canarina	**3**	13.8	8.2	**0.04944696**	0.04289480	0.01436378	**0.99432670**	0.79735980	0.68574300
Platycodoneae	90	41.9	29.1	**0.13081310**	0.12126410	0.07700898	**0.99869090**	0.84497210	0.88199630
Campanulaceae	2400	80.0	76.0	**0.09329048**	0.08951007	0.07148787	**0.99755980**	0.81627100	0.87301420
Angiosperms	262196	–	132.0	**0.08927046**	0.08709107	0.07668942	**0.99738120**	0.81420940	0.88150240
**Ci. canariense**	**3**	12.2	3.4	**0.11925440**	0.10345220	0.03464207	**0.55555560**	0.52082350	0.47325100
Cicer	30	17.5	12.2	**0.22197130**	0.20076620	0.10728060	**0.77895850**	0.66313190	0.71042020
Vicioid clade	860	33.0	32.1	0.18890300	0.17997310	**0.13749020**	0.72322220	0.63828420	**0.76513850**
Fabaceae	18860	71.0	61.0	0.15002710	0.14531170	**0.12280980**	0.63947140	0.59148560	**0.74062200**
Angiosperms	262196	–	132.0	0.08927046	0.08709107	**0.07668942**	0.45503790	0.48584890	**0.63385480**
Co. schimperianum	**5**	6.3	4.9	**0.18699810**	0.15998920	0.05691240	**0.47520000**	0.44385090	0.41688380
Colchicum	160	32.3	25.5	0.17184420	0.16077950	**0.10885740**	0.42269740	0.44584400	**0.62769770**
Colchiceae	185	48.0	43.3	0.10455450	0.09802111	**0.06729433**	0.18023150	0.26406810	**0.46976100**
Colchicaceae	280	86.5	67.3	0.07342708	0.06919952	**0.04921930**	0.08535675	0.16504810	**0.37322020**
Angiosperms	262196	–	132.0	0.08927046	0.08709107	**0.07668942**	0.13062990	0.22741510	**0.51230800**
Eu. schimperiana	20	8.2	7.1	**0.32430780**	0.28984410	0.14255010	**0.42026500**	0.39358480	0.37850870
Esula	96	11.0	8.5	**0.45543540**	0.42267230	0.27056700	**0.82890250**	0.71703890	0.73821350
Euphorbia	2000	54.7	47.8	0.14451370	0.13850460	**0.10986400**	*0.00252786*	*0.02451810*	**0.24556170**
Euphorbiaceae	6600	69.2	64.6	0.12541300	0.12096180	**0.09972609**	*0.00062042*	*0.01108944*	**0.20327080**

				bd.ms			crown.p		
**Lineage**	**Richness**	**Stem**	**Crown**	**0.0**	**0.5**	**0.9**	**0.0**	**0.5**	**0.9**
Angiosperms	262196	–	132.0	0.08927046	0.08709107	**0.07668942**	*0.00001293*	*0.00108248*	**0.11175160**
**E. balsamifera**	**3**	7.2	3.8	**0.10670130**	0.09256247	0.03099554	**0.55555560**	0.52082350	0.47325100
E. omariana	30	8.2	7.4	**0.36595270**	0.33099290	0.17686810	**0.41534300**	0.38914730	0.37501580
Anthacanthae (+*Balsamis*)	170	11.9	10.8	**0.41135660**	0.38520160	0.26234810	**0.95612060**	0.84510890	0.90099900
Euphorbia	2000	54.7	47.8	0.14451370	0.13850460	**0.10986400**	0.66656400	0.60508700	**0.74058510**
Euphorbiaceae	6600	69.2	64.6	0.12541300	0.12096180	**0.09972609**	0.61447090	0.57617230	**0.71903000**
Angiosperms	262196	N/A	132.0	0.08927046	0.08709107	**0.07668942**	0.49259990	0.50841650	**0.65921960**
Eu. usambarica	12	5.6	4.4	**0.40721810**	0.35776220	0.15644910	**0.43068160**	0.40300640	0.38586230
Eu. tuckeyana	23	9.3	5.6	**0.43613340**	0.39146610	0.19834370	**0.41831760**	0.39182810	0.37712820
Aphyllis + *Exiguae* II	26	12.8	10.4	0.24662970	0.22217220	**0.11543010**	*0.01439753*	*0.04916386*	**0.12601220**
Euphorbia	2000	54.7	47.8	0.14451370	0.13850460	**0.10986400**	*0.00004386*	*0.00222117*	**0.11074760**
Euphorbiaceae	6600	69.2	64.6	0.12541300	0.12096180	**0.09972609**	*0.00000658*	*0.00071004*	**0.08463664**
Angiosperms	262196	N/A	132.0	0.08927046	0.08709107	**0.07668942**	*0.00000004*	*0.00002784*	***0.03636881***
G. robertianum	20	11.0	8.1	**0.28426980**	0.25406090	0.12495130	**0.42026500**	0.39358480	0.37850870
Robertium	30	15.3	11.0	0.24618640	0.22266800	**0.11898400**	0.28105320	0.30437140	**0.34601080**
Geranium	430	26.4	15.3	0.35102210	0.33235420	**0.24382760**	0.75725780	0.67324010	**0.76906770**
Geraniaceae	800	56.0	39.0	0.15362730	0.14627930	**0.11133050**	*0.01250196*	0.05990260	**0.30877360**
Angiosperms	262196	N/A	132.0	0.08927046	0.08709107	**0.07668942**	*0.00002672*	*0.00193325*	**0.13973240**
H. quartinianum	**2.01**	4.0	1.3	**0.00383657**	0.00381790	0.00297116	**0.95778500**	0.95494310	0.91589870
Campylosporus	**5**	16.0	4.0	**0.22907270**	0.19598680	0.06971769	**0.99385050**	0.79998560	0.68437510
H. scopulorum	**10**	21.0	17.3	**0.09303109**	0.08120901	0.03397611	**0.43620760**	0.40802270	0.38974430
Androsaemum	15	27.1	21.0	**0.09594776**	0.08494803	0.03916164	**0.46646460**	0.43687500	0.45206240
Hypericum	500	49.9	34.9	0.15820800	0.15001590	**0.11113240**	0.49518800	0.50072870	**0.70362740**
Hypericaceae	585	61.0	53.9	0.10535180	0.10004270	**0.07482297**	0.16194090	0.25492070	**0.53140710**
Angiosperms	262196	N/A	132.0	0.08927046	0.08709107	**0.07668942**	0.08694648	0.18865990	**0.54252850**
K. neriifolia	**10**	11.2	6.8	0.23668200	0.20660530	0.08643922	**0.43620760**	0.40802270	0.38974430

Lineage	Richness	Stem	Crown	bd.ms			crown.p		
				**0.0**	**0.5**	**0.9**	**0.0**	**0.5**	**0.9**
Kleinia	60	14.8	11.8	0.28823710	0.26509890	**0.15930560**	0.63133870	0.57122420	**0.66096100**
Senecioneae	3000	27.0	25.5	0.28679300	0.27552290	**0.22178340**	0.62647160	0.59647250	**0.79029560**
Asteraceae	23000	44.0	40.0	0.23375260	0.22656150	**0.19224400**	0.42414780	0.46759840	**0.73754260**
Angiosperms	262196	N/A	132.0	0.08927046	0.08709107	**0.07668942**	*0.00985193*	0.05161934	**0.33947270**
P. crocyllis	**9**	6.2	3.7	0.40650740	0.35354310	0.14415430	**0.44002160**	0.41149290	0.39241680
P. pendula	**10**	9.5	6.2	0.25958680	0.22659930	0.09480430	**0.43620760**	0.40802270	0.38974430
Plocama	34	22.7	10.2	0.27776600	0.25207530	**0.13789870**	0.50303780	0.47700480	**0.55683810**
Putorieae	36	34.0	31.9	0.09060727	0.08234886	**0.04551950**	*0.00647617*	*0.03079895*	**0.13585340**
Rubiaceae	13600	65.0	57.0	0.15481890	0.14977300	**0.12569480**	0.08541263	0.18312110	**0.51502430**
Angiosperms	262196	N/A	132.0	0.08927046	0.08709107	**0.07668942**	*0.00596619*	*0.03764727*	**0.30256190**
S. spinosus	**2.01**	47.3	17.5	**0.00028500**	0.00028362	0.00022071	**0.95778500**	0.95494310	0.91589870
African clade	20	50.5	47.3	**0.04868045**	0.04350725	0.02139758	**0.99869610**	0.84122420	0.84539730
Sideroxylon	80	74.5	56.3	**0.06552184**	0.06060775	0.03791194	**0.99934930**	0.88020500	0.91303010
Sapotaceae	800	77.0	74.5	**0.08042234**	0.07657574	0.05828039	**0.99963740**	0.90931830	0.95039740
Angiosperms	262196	–	132.0	**0.08927046**	0.08709107	0.07668942	**0.99974150**	0.92471130	0.96791170

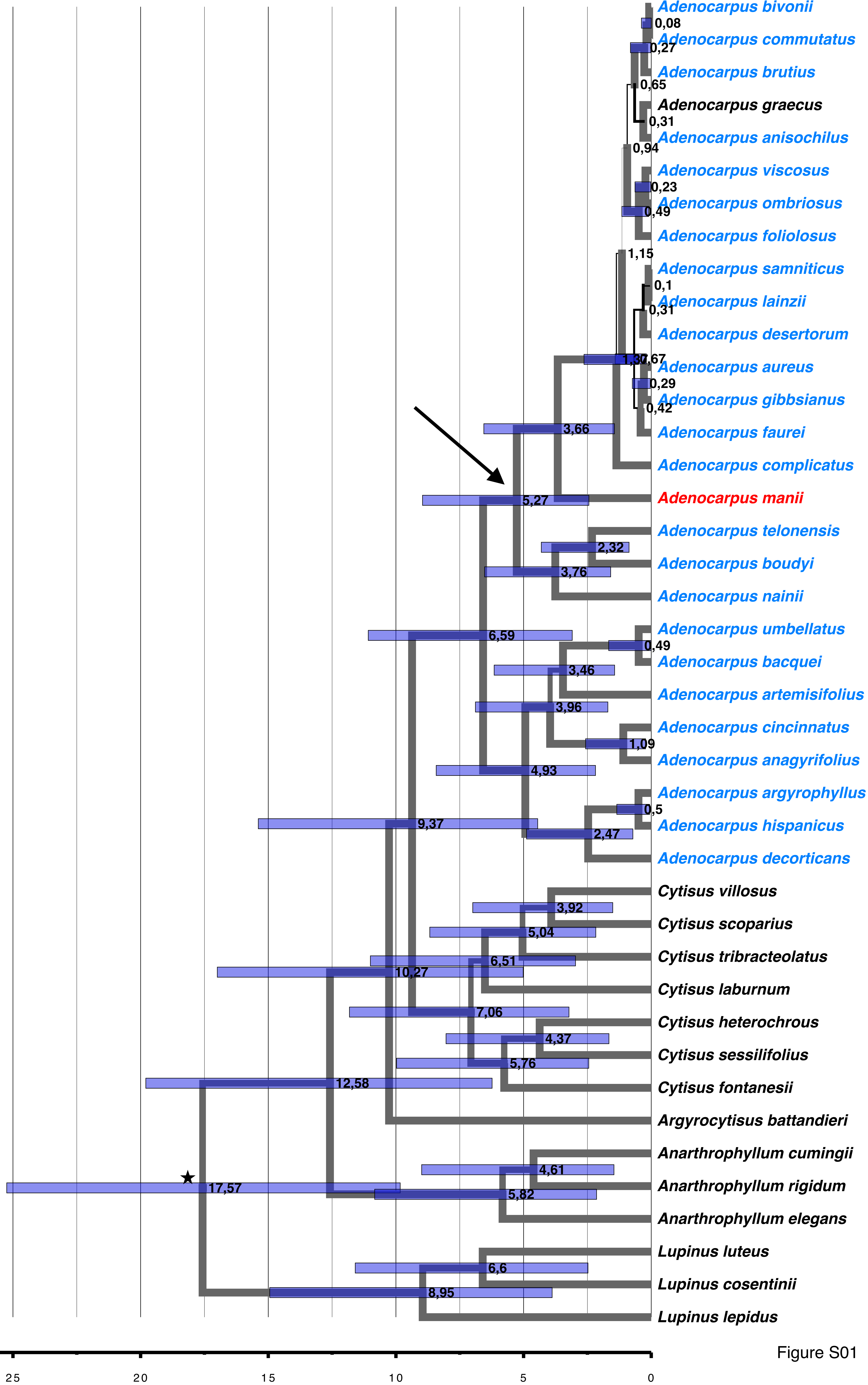
Adenocarpus bivonii
0,08
Adenocarpus commutatus
0,27
Adenocarpus brutius
0,65
Adenocarpus graecus
0,31
Adenocarpus anisochilus
0,94
Adenocarpus viscosus
0,23
Adenocarpus ombriosus
0,49
Adenocarpus foliolosus
1,15
Adenocarpus samniticus
0,1
Adenocarpus lainzii
0,31
Adenocarpus desertorum
0,67
Adenocarpus aureus
0,29
Adenocarpus gibbsianus
0,42
3,66
Adenocarpus faurei
Adenocarpus complicatus
Adenocarpus manii
5,27
Adenocarpus telonensis
2,32
Adenocarpus boudyi
3,76
Adenocarpus nainii
Adenocarpus umbellatus
6,59
0,49
Adenocarpus bacquei
3,46
Adenocarpus artemisifolius
3,96
Adenocarpus cincinnatus
1,09
Adenocarpus anagyrifolius
4,93
Adenocarpus argyrophyllus
0,5
9,37
Adenocarpus hispanicus
2,47
Adenocarpus decorticans
Cytisus villosus
3,92
Cytisus scoparius
5,04
Cytisus tribracteolatus
6,51
10,27
Cytisus laburnum
7,06
Cytisus heterochrous
4,37
Cytisus sessilifolius
5,76
12,58
Cytisus fontanesii
Argyrocytisus battandieri
Anarthrophyllum cumingii
4,61
17,57
Anarthrophyllum rigidum
5,82
Anarthrophyllum elegans
Lupinus luteus
6,6
Lupinus cosentinii
8,95
Lupinus lepidus
25
20
15
10
5
0

Figure S01

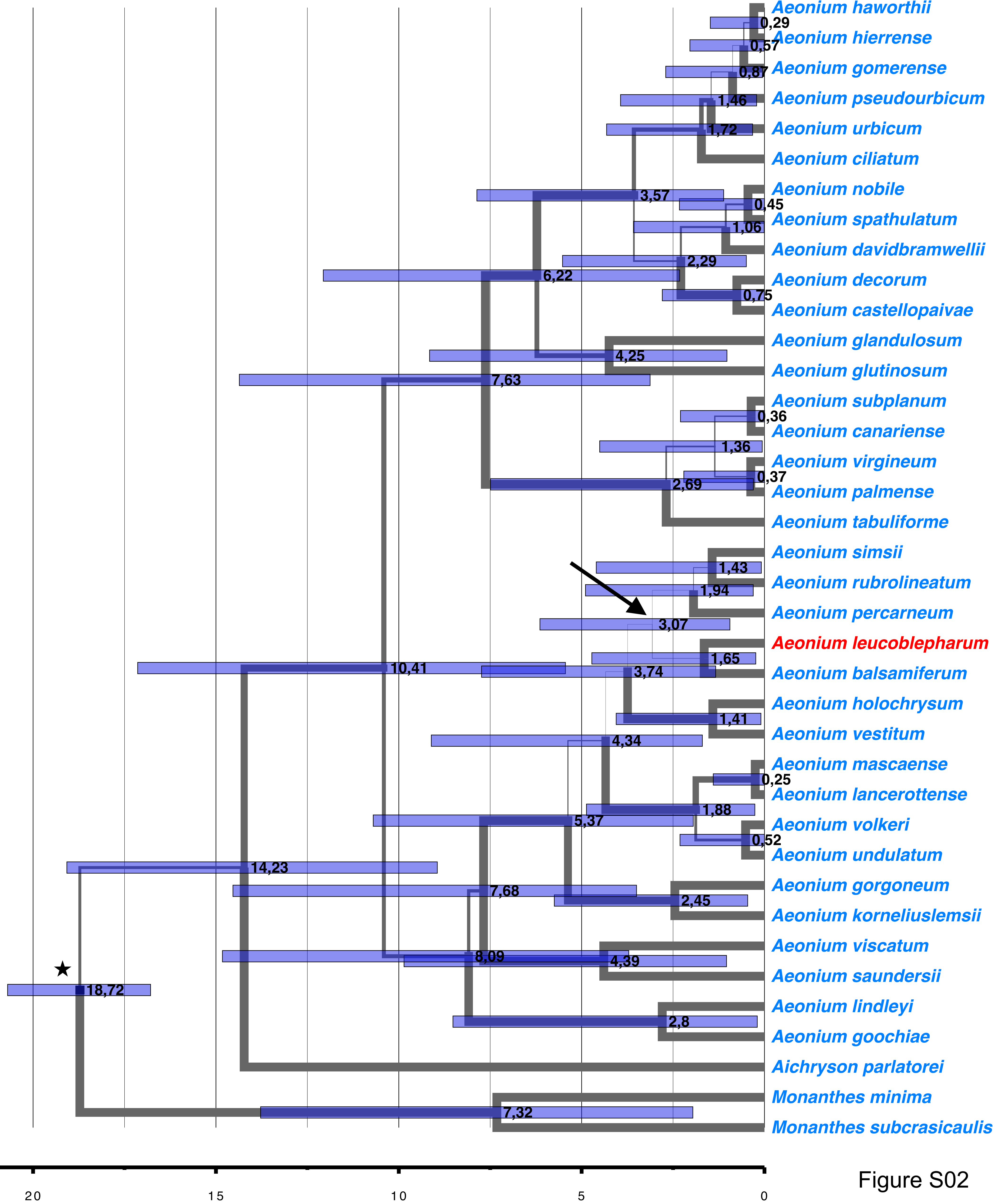
Aeonium haworthii
Aeonium hierrense
Aeonium gomerense
Aeonium pseudourbicum
Aeonium urbicum
Aeonium ciliatum
Aeonium nobile
Aeonium spathulatum
Aeonium davidbramwellii
Aeonium decorum
Aeonium castellopaivae
Aeonium glandulosum
Aeonium glutinosum
Aeonium subplanum
Aeonium canariense
Aeonium virgineum
Aeonium palmense
Aeonium tabuliforme
Aeonium simsii
Aeonium rubrolineatum
Aeonium percarneum
Aeonium leucoblepharum
Aeonium balsamiferum
Aeonium holochrysum
Aeonium vestitum
Aeonium mascaense
Aeonium lancerottense
Aeonium volkeri
Aeonium undulatum
Aeonium gorgoneum
Aeonium korneliuslemsii
Aeonium viscatum
Aeonium saundersii
Aeonium lindleyi
Aeonium goochiae
Aichryson parlatorei
Monanthes minima
Monanthes subcrasicaulis
0,29
0,57
0,87
1,46
1,72
3,57
0,45
1,06
2,29
6,22
0,75
4,25
7,63
0,36
1,36
0,37
2,69
1,43
1,94
3,07
1,65
10,41
3,74
1,41
4,34
0,25
1,88
5,37
0,52
14,23
7,68
2,45
8,09
4,39
18,72
2,8
7,32
20
15
10
5
0

Figure S02

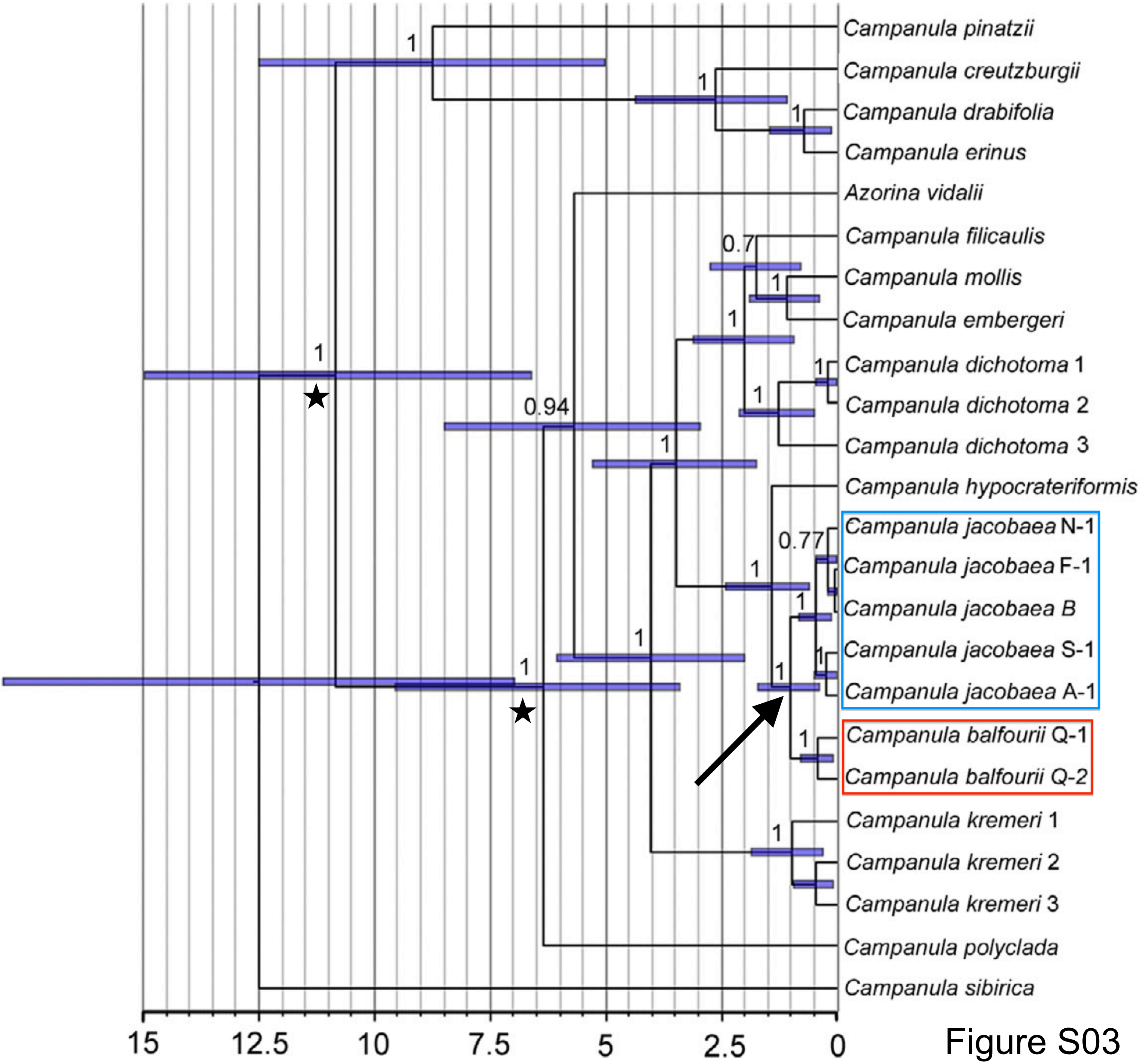
Campanula pinatzii
Campanula creutzburgii
Campanula drabifolia
Campanula erinus
Azorina vidalii
Campanula filicaulis
Campanula mollis
Campanula embergeri
Campanula dichotoma 1
Campanula dichotoma 2
Campanula dichotoma 3
Campanula hypocrateriformis
Campanula jacobaea N-1
Campanula jacobaea F-1
Campanula jacobaea B
Campanula jacobaea S-1
Campanula jacobaea A-1
Campanula balfourii Q-1
Campanula balfourii Q-2
Campanula kremeri 1
Campanula kremeri 2
Campanula kremeri 3
Campanula polyclada
Campanula sibirica
1
1
1
0.7
1
1
1
1
0.94
1
1
0.77
1
1
1
1
1
1
15
12.5
10
7.5
5
2.5
0
Figure S03

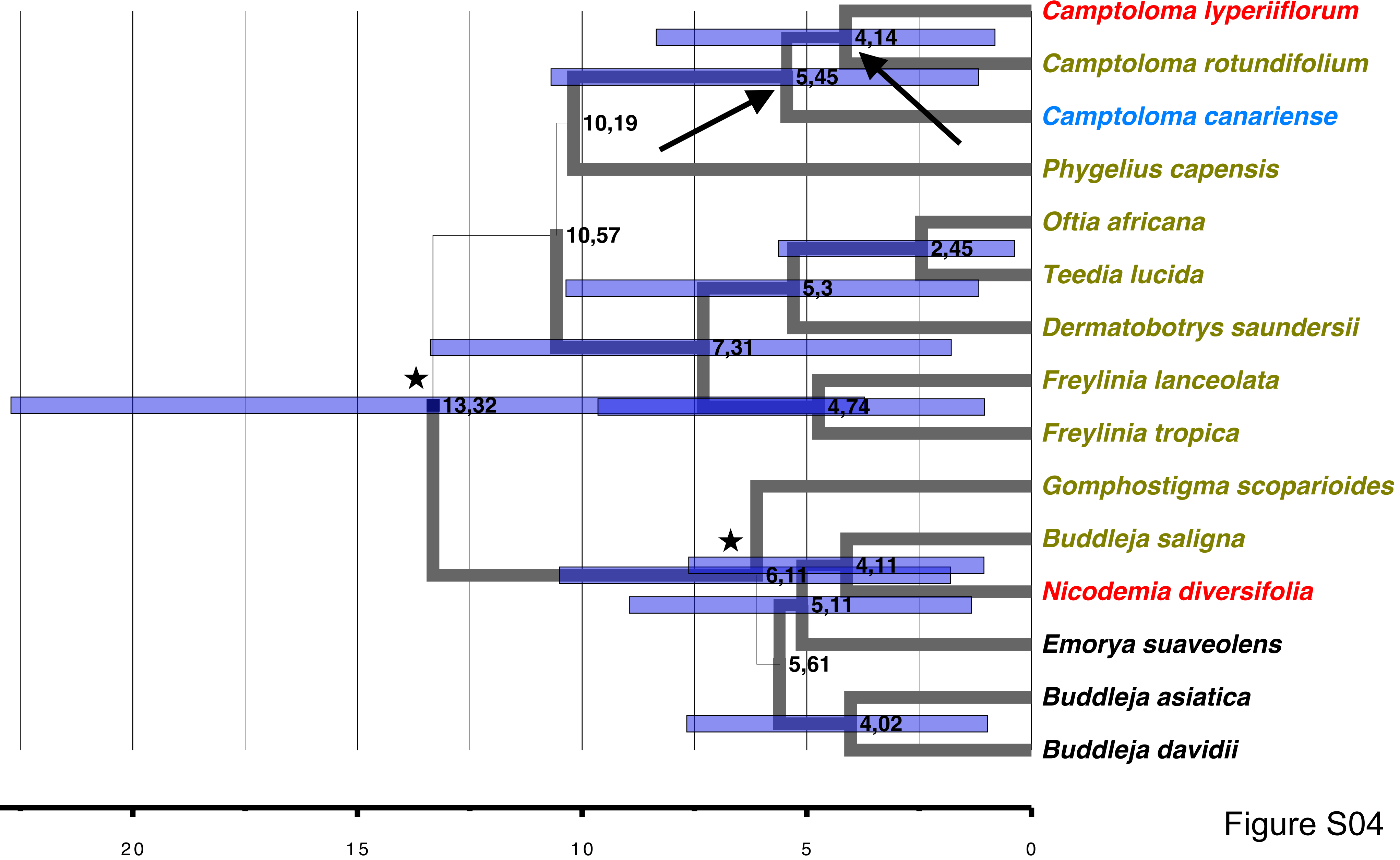
Camptoloma lyperiiflorum
Camptoloma rotundifolium
Camptoloma canariense
Phygelius capensis
Oftia africana
Teedia lucida
Dermatobotrys saundersii
Freylinia lanceolata
Freylinia tropica
Gomphostigma scoparioides
Buddleja saligna
Nicodemia diversifolia
Emorya suaveolens
Buddleja asiatica
Buddleja davidii
4,14
5,45
10,19
10,57
2,45
5,3
7,31
13,32
4,74
4,11
6,11
5,11
5,61
4,02
20
15
10
5
0

Figure S04

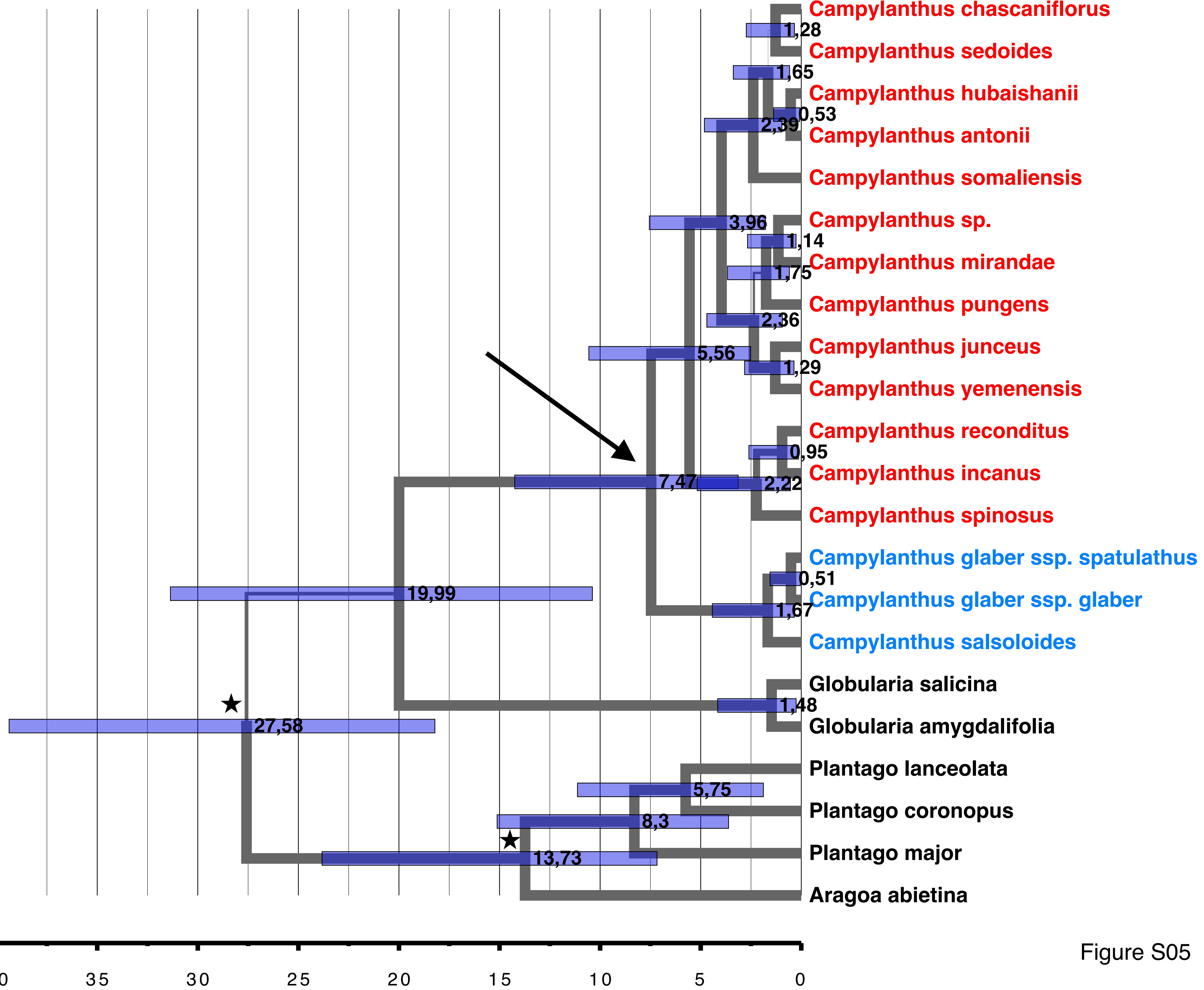
Campylanthus chascaniflorus
1,28
Campylanthus sedoides
1,65
Campylanthus hubaishanii
0,53
2,39
Campylanthus antonii
Campylanthus somaliensis
3,96
Campylanthus sp.
1,14
Campylanthus mirandae
1,75
Campylanthus pungens
2,36
Campylanthus junceus
5,56
1,29
Campylanthus yemenensis
Campylanthus reconditus
0,95
Campylanthus incanus
7,47
2,22
Campylanthus spinosus
Campylanthus glaber ssp. spatulathus
0,51
19,99
Campylanthus glaber ssp. glaber
1,67
Campylanthus salsoloides
Globularia salicina
1,48
27,58
Globularia amygdalifolia
Plantago lanceolata
5,75
Plantago coronopus
8,3
13,73
Plantago major
Aragoa abietina
0
35
30
25
20
15
10
5
0

Figure S05

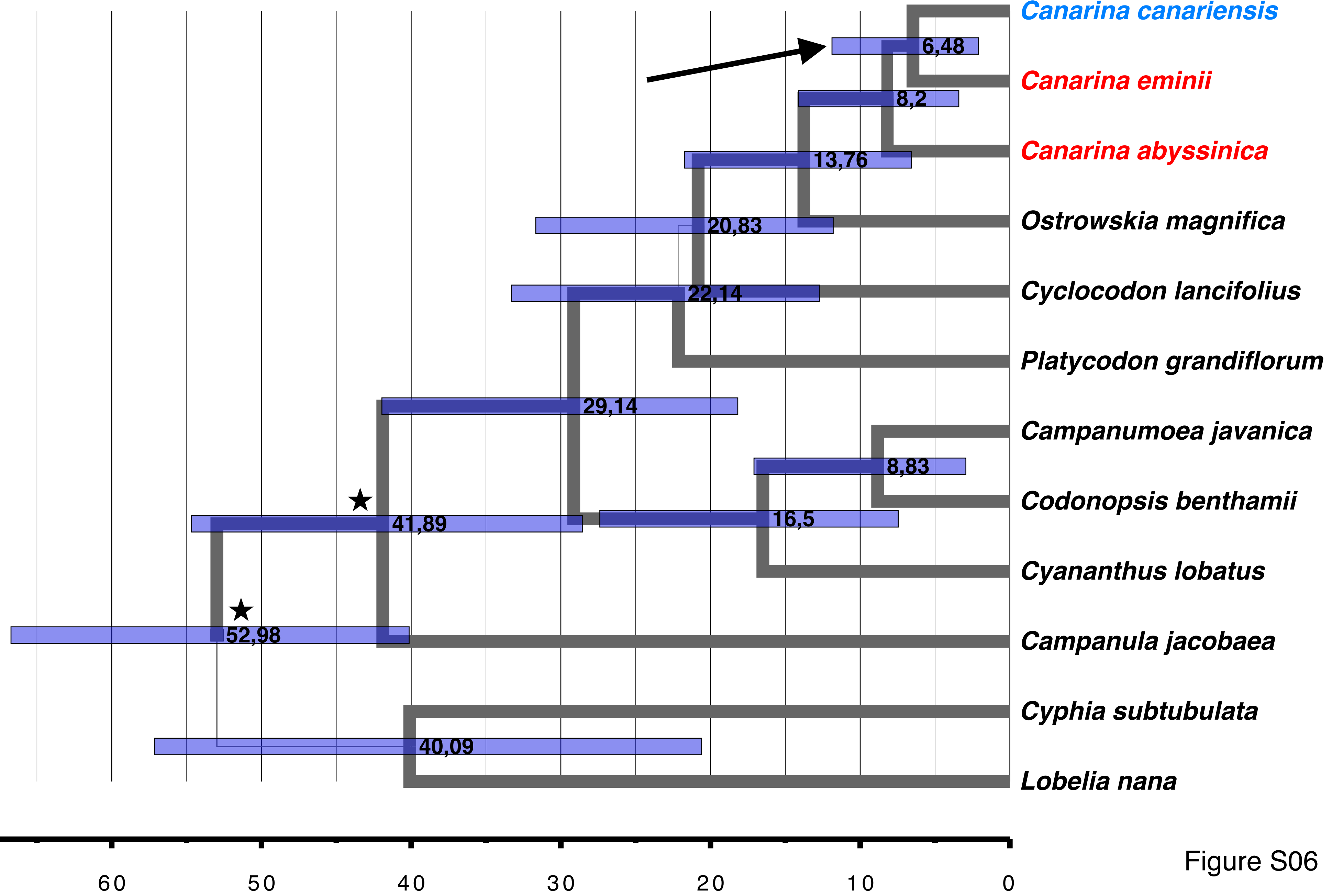

Canarina canariensis
Canarina eminii
Canarina abyssinica
Ostrowskia magnifica
Cyclocodon lancifolius
Platycodon grandiflorum
Campanumoea javanica
Codonopsis benthamii
Cyananthus lobatus
Campanula jacobaea
Cyphia subtubulata
Lobelia nana
6,48
8,2
13,76
20,83
22,14
29,14
8,83
41,89
16,5
52,98
40,09
60
50
40
30
20
10
0

Figure S06

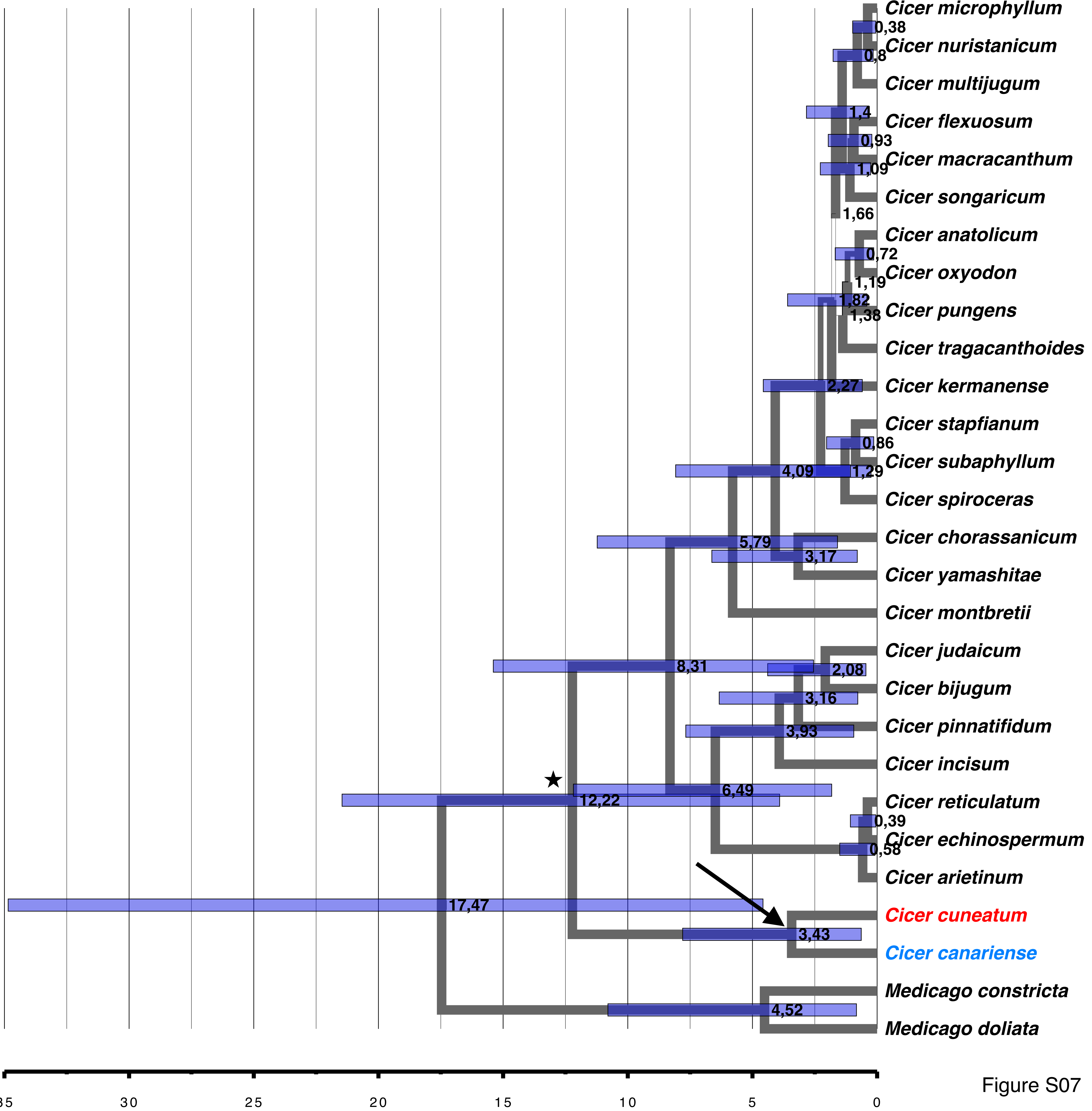
Cicer microphyllum
0,38
Cicer nuristanicum
0,8
Cicer multijugum
1,4
Cicer flexuosum
0,93
Cicer macracanthum
1,09
Cicer songaricum
1,66
Cicer anatolicum
0,72
Cicer oxyodon
1,19
1,82
Cicer pungens
1,38
Cicer tragacanthoides
2,27
Cicer kermanense
Cicer stapfianum
0,86
Cicer subaphyllum
4,09
1,29
Cicer spiroceras
Cicer chorassanicum
5,79
3,17
Cicer yamashitae
Cicer montbretii
Cicer judaicum
8,31
2,08
Cicer bijugum
3,16
Cicer pinnatifidum
3,93
Cicer incisum
6,49
12,22
Cicer reticulatum
0,39
Cicer echinospermum
0,58
Cicer arietinum
17,47
Cicer cuneatum
3,43
Cicer canariense
Medicago constricta
4,52
Medicago doliata
35
30
25
20
15
10
5
0

Figure S07

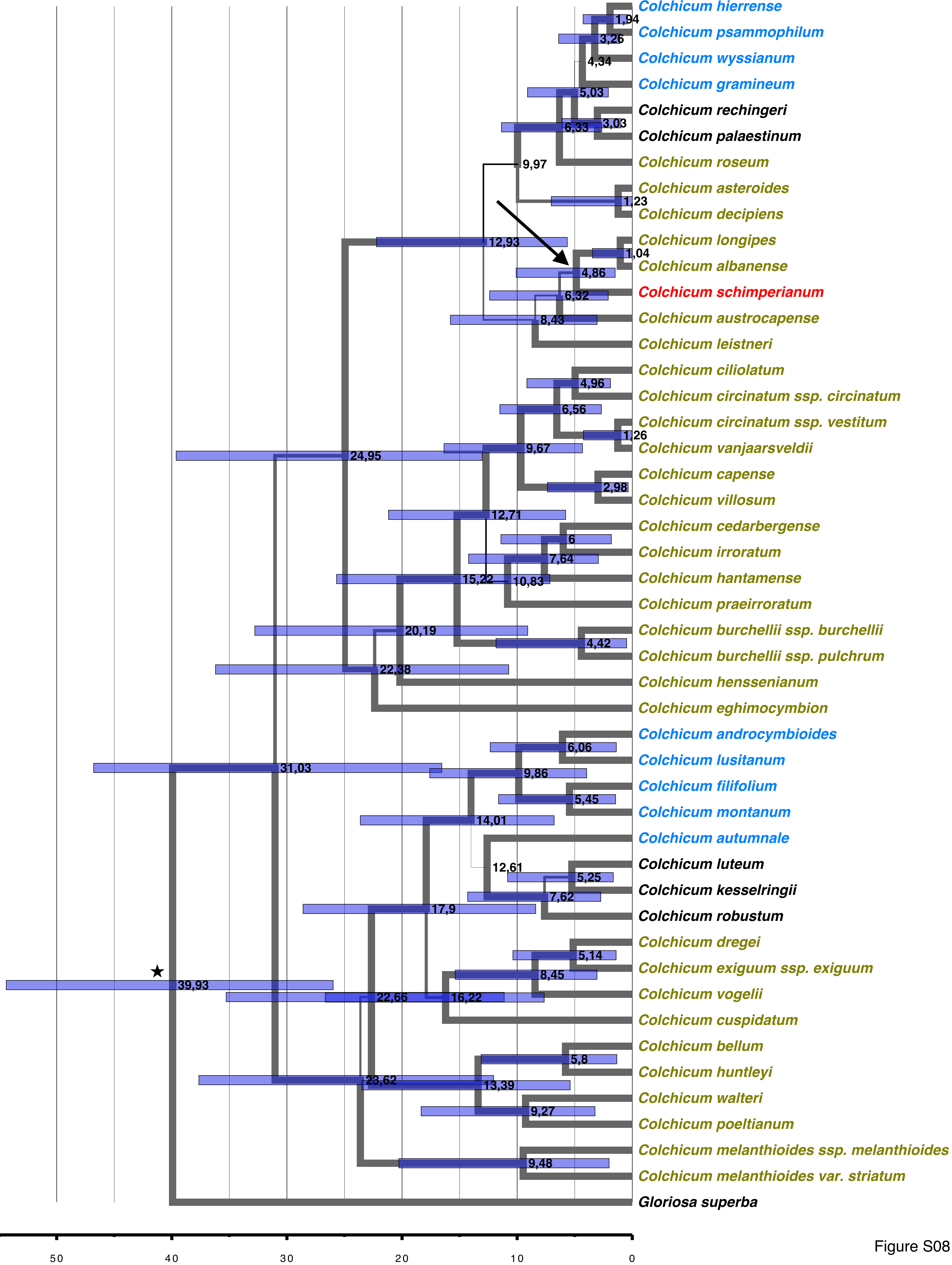

Colchicum hierrense
1,94
Colchicum psammophilum
3,26
Colchicum wyssianum
4,34
Colchicum gramineum
5,03
Colchicum rechingeri
3,03
6,33
Colchicum palaestinum
Colchicum roseum
9,97
Colchicum asteroides
1,23
Colchicum decipiens
Colchicum longipes
12,93
1,04
Colchicum albanense
4,86
Colchicum schimperianum
6,32
Colchicum austrocapense
8,43
Colchicum leistneri
Colchicum ciliolatum
4,96
Colchicum circinatum ssp. circinatum
6,56
Colchicum circinatum ssp. vestitum
1,26
24,95
9,67
Colchicum vanjaarsveldii
Colchicum capense
2,98
Colchicum villosum
12,71
Colchicum cedarbergense
6
7,64
Colchicum irroratum
15,22
10,83
Colchicum hantamense
Colchicum praeirroratum
20,19
Colchicum burchellii ssp. burchellii
4,42
Colchicum burchellii ssp. pulchrum
22,38
Colchicum henssenianum
Colchicum eghimocymbion
Colchicum androcymbioides
6,06
31,03
Colchicum lusitanum
9,86
Colchicum filifolium
5,45
Colchicum montanum
14,01
Colchicum autumnale
Colchicum luteum
12,61
5,25
Colchicum kesselringii
7,62
17,9
Colchicum robustum
Colchicum dregei
5,14
Colchicum exiguum ssp. exiguum
8,45
39,93
Colchicum vogelii
22,66
16,22
Colchicum cuspidatum
Colchicum bellum
5,8
Colchicum huntleyi
23,62
13,39
Colchicum walteri
9,27
Colchicum poeltianum
Colchicum melanthioides ssp. melanthioides
9,48
Colchicum melanthioides var. striatum
Gloriosa superba
50
40
30
20
10
0

Figure S08

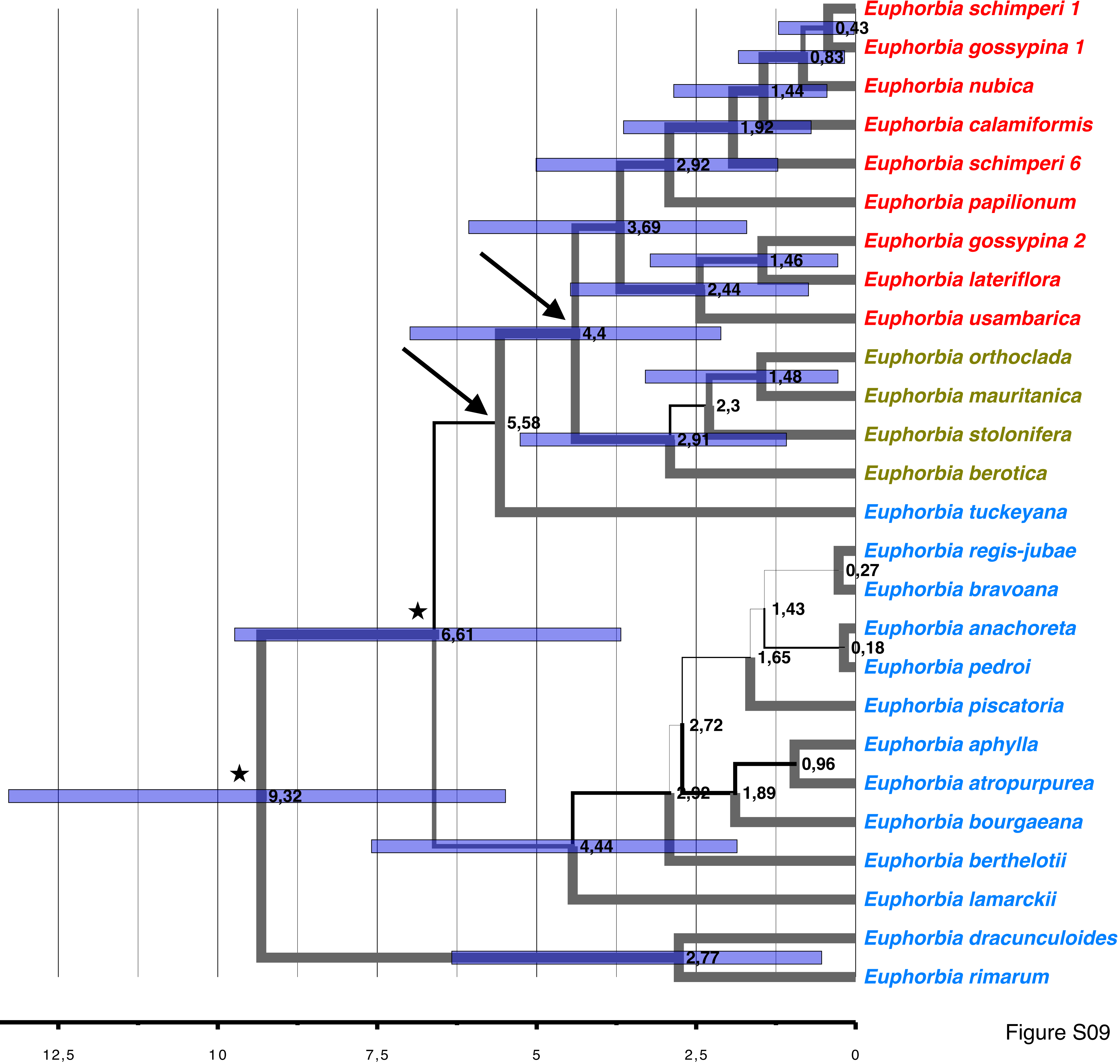

Euphorbia schimperi 1
Euphorbia gossypina 1
Euphorbia nubica
Euphorbia calamiformis
Euphorbia schimperi 6
Euphorbia papilionum
Euphorbia gossypina 2
Euphorbia lateriflora
Euphorbia usambarica
Euphorbia orthoclada
Euphorbia mauritanica
Euphorbia stolonifera
Euphorbia berotica
Euphorbia tuckeyana
Euphorbia regis-jubae
Euphorbia bravoana
Euphorbia anachoreta
Euphorbia pedroi
Euphorbia piscatoria
Euphorbia aphylla
Euphorbia atropurpurea
Euphorbia bourgaeana
Euphorbia berthelotii
Euphorbia lamarckii
Euphorbia dracunculoides
Euphorbia rimarum
0,43
0,83
1,44
1,92
2,92
3,69
1,46
2,44
4,4
1,48
2,3
5,58
2,91
0,27
1,43
6,61
0,18
1,65
2,72
0,96
9,32
2,92
1,89
4,44
2,77
12,5
10
7,5
5
2,5
0

Figure S09

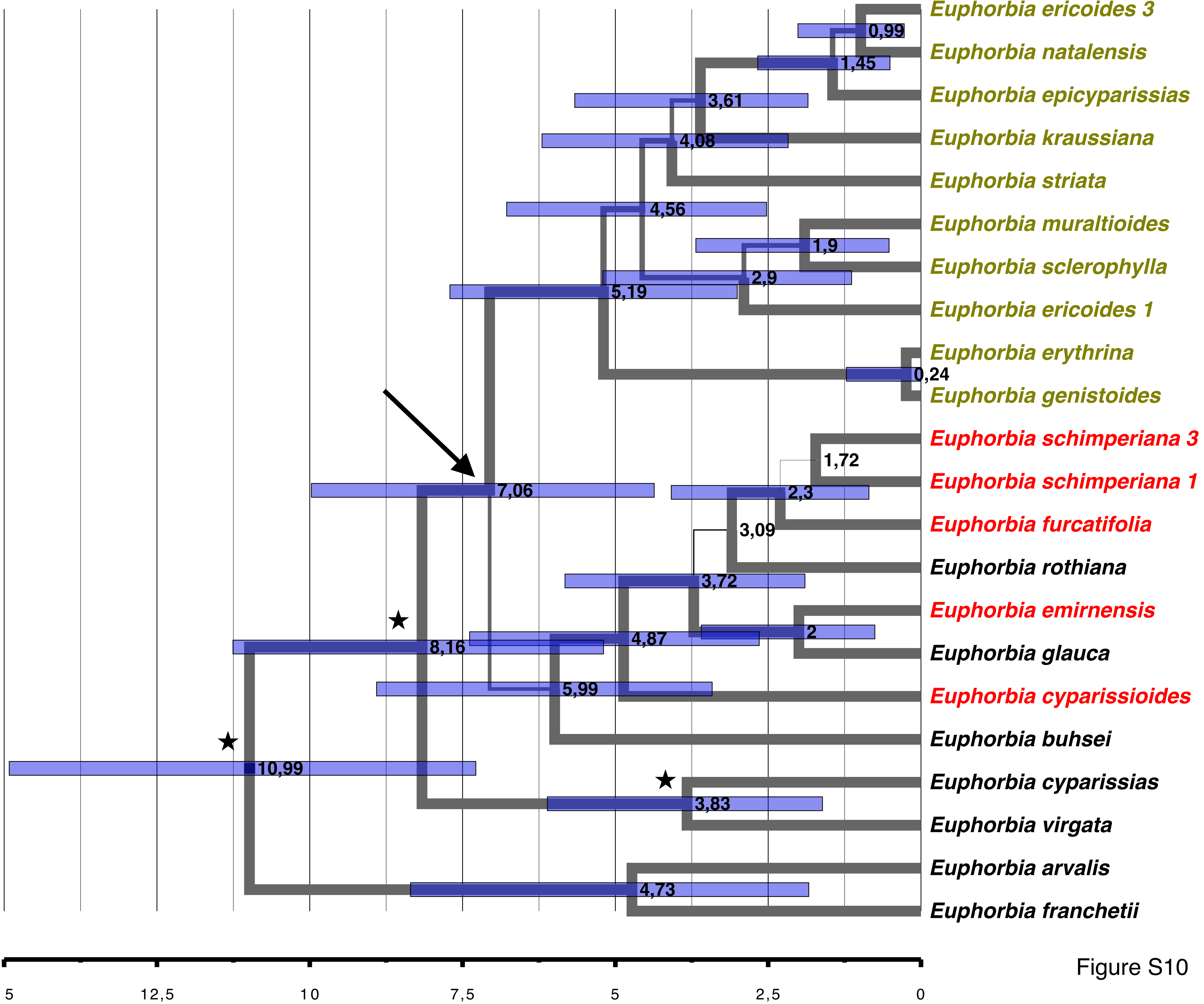

Euphorbia ericoides 3
Euphorbia natalensis
Euphorbia epicyparissias
Euphorbia kraussiana
Euphorbia striata
Euphorbia muraltioides
Euphorbia sclerophylla
Euphorbia ericoides 1
Euphorbia erythrina
Euphorbia genistoides
Euphorbia schimperiana 3
Euphorbia schimperiana 1
Euphorbia furcatifolia
Euphorbia rothiana
Euphorbia emirnensis
Euphorbia glauca
Euphorbia cyparissioides
Euphorbia buhsei
Euphorbia cyparissias
Euphorbia virgata
Euphorbia arvalis
Euphorbia franchetii
0,99
1,45
3,61
4,08
4,56
1,9
2,9
5,19
0,24
1,72
7,06
2,3
3,09
3,72
2
8,16
4,87
5,99
10,99
3,83
4,73
5
12,5
10
7,5
5
2,5
0

Figure S10

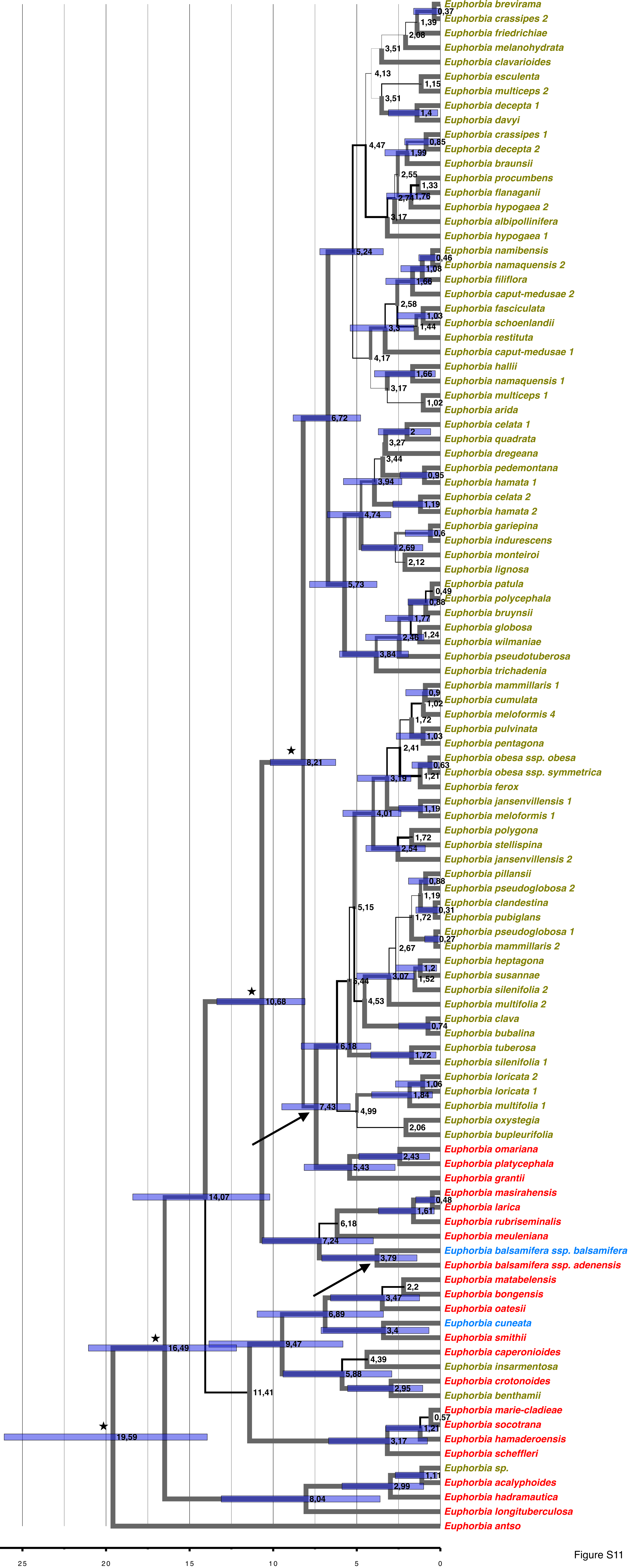
Euphorbia brevirama
Euphorbia crassipes 2
Euphorbia friedrichiae
Euphorbia melanohydrata
Euphorbia clavarioides
Euphorbia esculenta
Euphorbia multiceps 2
Euphorbia decepta 1
Euphorbia davyi
Euphorbia crassipes 1
Euphorbia decepta 2
Euphorbia braunsii
Euphorbia procumbens
Euphorbia flanaganii
Euphorbia hypogaea 2
Euphorbia albipollinifera
Euphorbia hypogaea 1
Euphorbia namibensis
Euphorbia namaquensis 2
Euphorbia filiflora
Euphorbia caput-medusae 2
Euphorbia fasciculata
Euphorbia schoenlandii
Euphorbia restituta
Euphorbia caput-medusae 1
Euphorbia hallii
Euphorbia namaquensis 1
Euphorbia multiceps 1
Euphorbia arida
Euphorbia celata 1
Euphorbia quadrata
Euphorbia dregeana
Euphorbia pedemontana
Euphorbia hamata 1
Euphorbia celata 2
Euphorbia hamata 2
Euphorbia gariepina
Euphorbia indurescens
Euphorbia monteiroi
Euphorbia lignosa
Euphorbia patula
Euphorbia polycephala
Euphorbia bruynsii
Euphorbia globosa
Euphorbia wilmaniae
Euphorbia pseudotuberosa
Euphorbia trichadenia
Euphorbia mammillaris 1
Euphorbia cumulata
Euphorbia meloformis 4
Euphorbia pulvinata
Euphorbia pentagona
Euphorbia obesa ssp. obesa
Euphorbia obesa ssp. symmetrica
Euphorbia ferox
Euphorbia jansenvillensis 1
Euphorbia meloformis 1
Euphorbia polygona
Euphorbia stellispina
Euphorbia jansenvillensis 2
Euphorbia pillansii
Euphorbia pseudoglobosa 2
Euphorbia clandestina
Euphorbia pubiglans
Euphorbia pseudoglobosa 1
Euphorbia mammillaris 2
Euphorbia heptagona
Euphorbia susannae
Euphorbia silenifolia 2
Euphorbia multifolia 2
Euphorbia clava
Euphorbia bubalina
Euphorbia tuberosa
Euphorbia silenifolia 1
Euphorbia loricata 2
Euphorbia loricata 1
Euphorbia multifolia 1
Euphorbia oxystegia
Euphorbia bupleurifolia
Euphorbia omariana
Euphorbia platycephala
Euphorbia grantii
Euphorbia masirahensis
Euphorbia larica
Euphorbia rubriseminalis
Euphorbia meuleniana
Euphorbia balsamifera ssp. balsamifera
Euphorbia balsamifera ssp. adenensis
Euphorbia matabelensis
Euphorbia bongensis
Euphorbia oatesii
Euphorbia cuneata
Euphorbia smithii
Euphorbia caperonioides
Euphorbia insarmentosa
Euphorbia crotonoides
Euphorbia benthamii
Euphorbia marie-cladieae
Euphorbia socotrana
Euphorbia hamaderoensis
Euphorbia scheffleri
Euphorbia sp.
Euphorbia acalyphoides
Euphorbia hadramautica
Euphorbia longituberculosa
Euphorbia antso
25
20
15
10
5
0

Figure S11

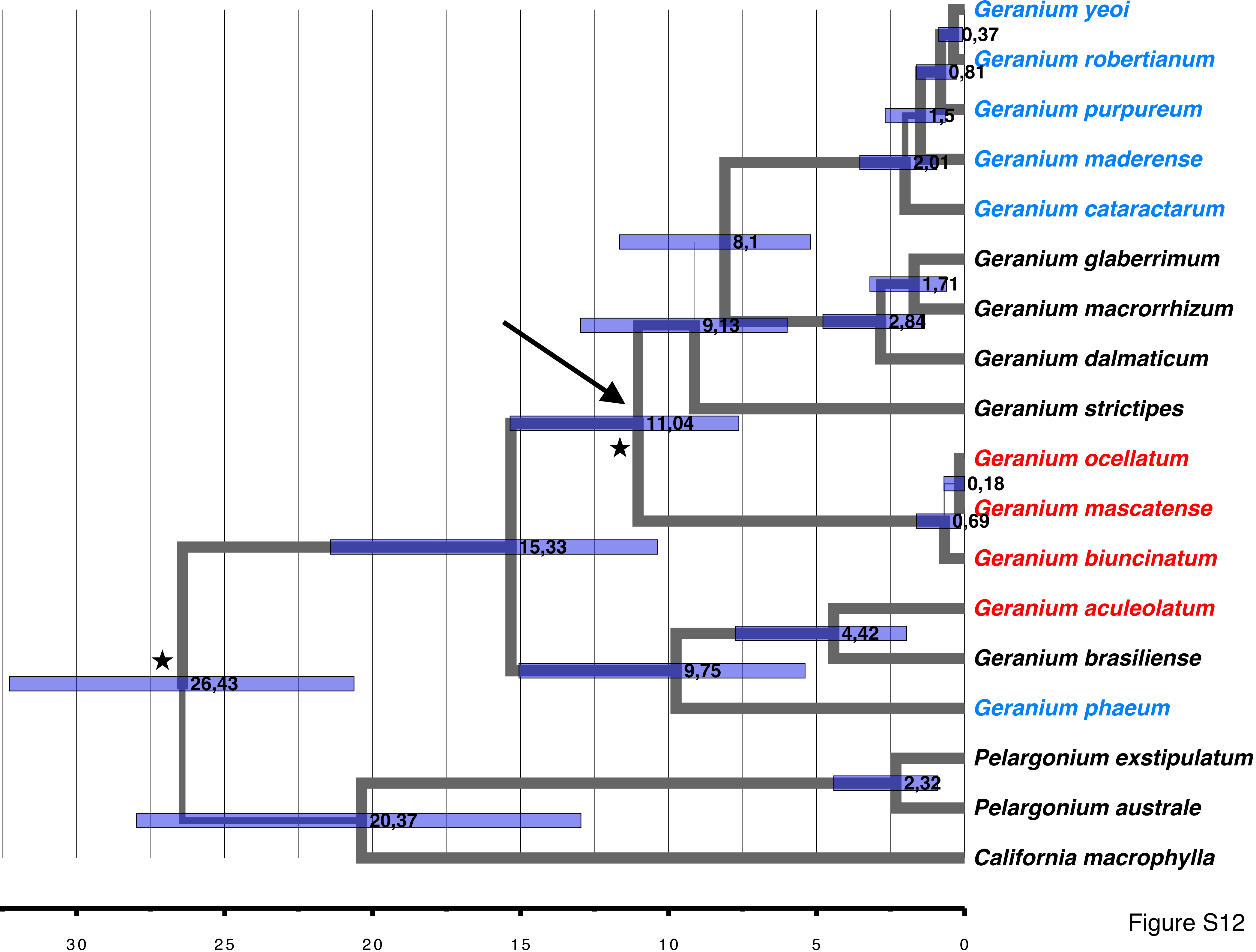

Geranium yeoi
0,37
Geranium robertianum
0,81
Geranium purpureum
1,5
Geranium maderense
2,01
Geranium cataractarum
8,1
Geranium glaberrimum
1,71
Geranium macrorrhizum
9,13
2,84
Geranium dalmaticum
Geranium strictipes
11,04
Geranium ocellatum
0,18
Geranium mascatense
0,69
15,33
Geranium biuncinatum
Geranium aculeolatum
4,42
Geranium brasiliense
26,43
9,75
Geranium phaeum
Pelargonium exstipulatum
2,32
Pelargonium australe
20,37
California macrophylla
30
25
20
15
10
5
0

Figure S12

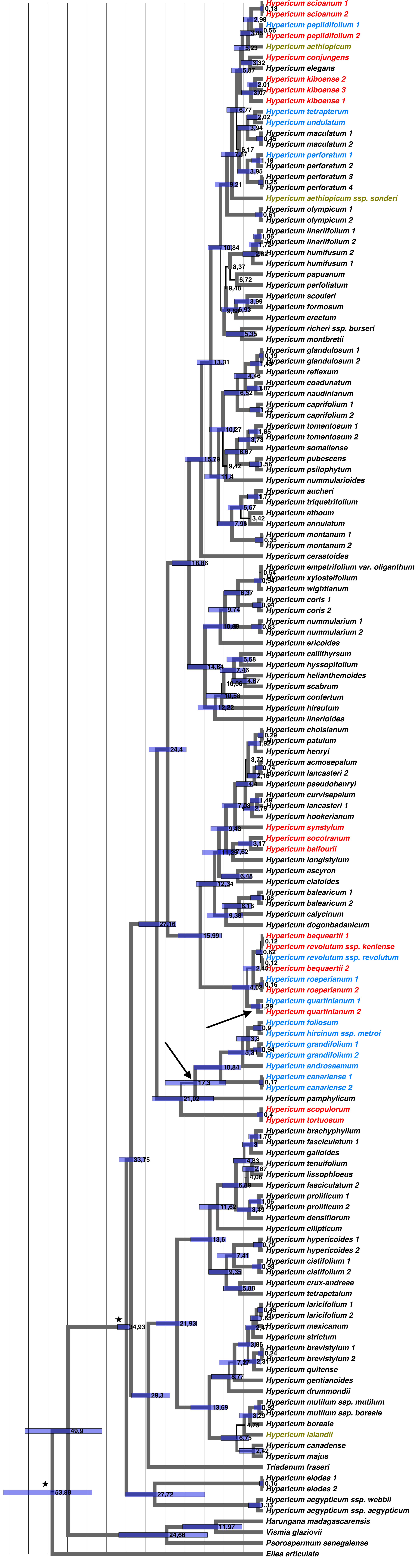
Hypericum scioanum 1
Hypericum scioanum 2
Hypericum peplidifolium 1
Hypericum peplidifolium 2
Hypericum aethiopicum
Hypericum conjungens
Hypericum elegans
Hypericum kiboense 2
Hypericum kiboense 3
Hypericum kiboense 1
Hypericum tetrapterum
Hypericum undulatum
Hypericum maculatum 1
Hypericum maculatum 2
Hypericum perforatum 1
Hypericum perforatum 2
Hypericum perforatum 3
Hypericum perforatum 4
Hypericum aethiopicum ssp. sonderi
Hypericum olympicum 1
Hypericum olympicum 2
Hypericum linariifolium 1
Hypericum linariifolium 2
Hypericum humifusum 2
Hypericum humifusum 1
Hypericum papuanum
Hypericum perfoliatum
Hypericum scouleri
Hypericum formosum
Hypericum erectum
Hypericum richeri ssp. burseri
Hypericum montbretii
Hypericum glandulosum 1
Hypericum glandulosum 2
Hypericum reflexum
Hypericum coadunatum
Hypericum naudinianum
Hypericum caprifolium 1
Hypericum caprifolium 2
Hypericum tomentosum 1
Hypericum tomentosum 2
Hypericum somaliense
Hypericum pubescens
Hypericum psilophytum
Hypericum nummularioides
Hypericum aucheri
Hypericum triquetrifolium
Hypericum athoum
Hypericum annulatum
Hypericum montanum 1
Hypericum montanum 2
Hypericum cerastoides
Hypericum empetrifolium var. oliganthum
Hypericum xylosteifolium
Hypericum wightianum
Hypericum coris 1
Hypericum coris 2
Hypericum nummularium 1
Hypericum nummularium 2
Hypericum ericoides
Hypericum callithyrsum
Hypericum hyssopifolium
Hypericum helianthemoides
Hypericum scabrum
Hypericum confertum
Hypericum hirsutum
Hypericum linarioides
Hypericum choisianum
Hypericum patulum
Hypericum henryi
Hypericum acmosepalum
Hypericum lancasteri 2
Hypericum pseudohenryi
Hypericum curvisepalum
Hypericum lancasteri 1
Hypericum hookerianum
Hypericum synstylum
Hypericum socotranum
Hypericum balfourii
Hypericum longistylum
Hypericum ascyron
Hypericum elatoides
Hypericum balearicum 1
Hypericum balearicum 2
Hypericum calycinum
Hypericum dogonbadanicum
Hypericum bequaertii 1
Hypericum revolutum ssp. keniense
Hypericum revolutum ssp. revolutum
Hypericum bequaertii 2
Hypericum roeperianum 1
Hypericum roeperianum 2
Hypericum quartinianum 1
Hypericum quartinianum 2
Hypericum foliosum
Hypericum hircinum ssp. metroi
Hypericum grandifolium 1
Hypericum grandifolium 2
Hypericum androsaemum
Hypericum canariense 1
Hypericum canariense 2
Hypericum pamphylicum
Hypericum scopulorum
Hypericum tortuosum
Hypericum brachyphyllum
Hypericum fasciculatum 1
Hypericum galioides
Hypericum tenuifolium
Hypericum lissophloeus
Hypericum fasciculatum 2
Hypericum prolificum 1
Hypericum prolificum 2
Hypericum densiflorum
Hypericum ellipticum
Hypericum hypericoides 1
Hypericum hypericoides 2
Hypericum cistifolium 1
Hypericum cistifolium 2
Hypericum crux-andreae
Hypericum tetrapetalum
Hypericum laricifolium 1
Hypericum laricifolium 2
Hypericum mexicanum
Hypericum strictum
Hypericum brevistylum 1
Hypericum brevistylum 2
Hypericum quitense
Hypericum gentianoides
Hypericum drummondii
Hypericum mutilum ssp. mutilum
Hypericum mutilum ssp. boreale
Hypericum boreale
Hypericum lalandii
Hypericum canadense
Hypericum majus
Triadenum fraseri
Hypericum elodes 1
Hypericum elodes 2
Hypericum aegypticum ssp. webbii
Hypericum aegypticum ssp. aegypticum
Harungana madagascarensis
Vismia glaziovii
Psorospermum senegalense
Eliea articulata
60
50
40
30
20
10
0

Figure S13

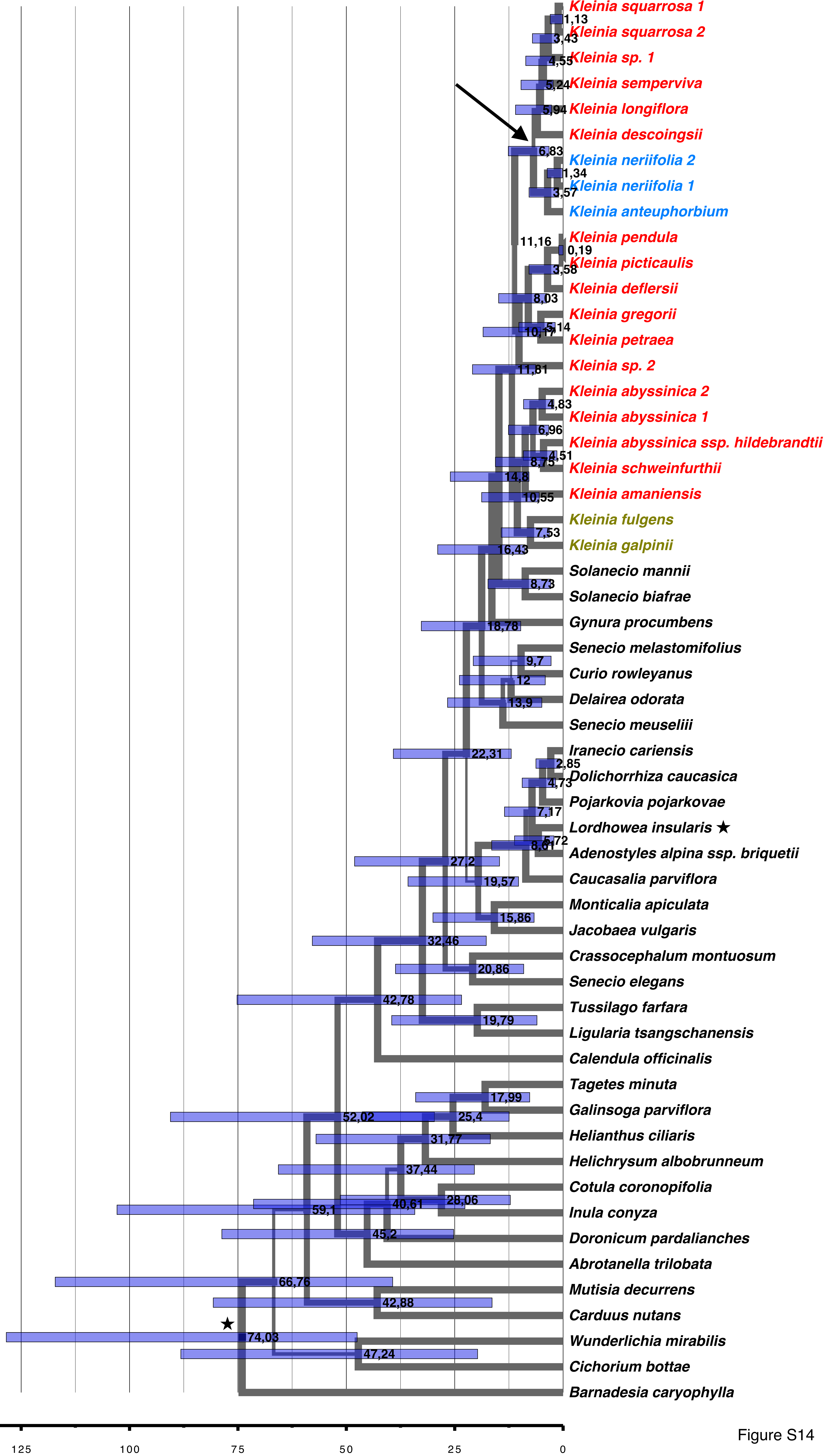

Kleinia squarrosa 1
Kleinia squarrosa 2
Kleinia sp. 1
Kleinia semperviva
Kleinia longiflora
Kleinia descoingsii
Kleinia neriifolia 2
Kleinia neriifolia 1
Kleinia anteuphorbium
Kleinia pendula
Kleinia picticaulis
Kleinia deflersii
Kleinia gregorii
Kleinia petraea
Kleinia sp. 2
Kleinia abyssinica 2
Kleinia abyssinica 1
Kleinia abyssinica ssp. hildebrandtii
Kleinia schweinfurthii
Kleinia amaniensis
Kleinia fulgens
Kleinia galpinii
Solanecio mannii
Solanecio biafrae
Gynura procumbens
Senecio melastomifolius
Curio rowleyanus
Delairea odorata
Senecio meuseliii
Iranecio cariensis
Dolichorrhiza caucasica
Pojarkovia pojarkovae
Lordhowea insularis ★
Adenostyles alpina ssp. briquetii
Caucasalia parviflora
Monticalia apiculata
Jacobaea vulgaris
Crassocephalum montuosum
Senecio elegans
Tussilago farfara
Ligularia tsangschanensis
Calendula officinalis
Tagetes minuta
Galinsoga parviflora
Helianthus ciliaris
Helichrysum albobrunneum
Cotula coronopifolia
Inula conyza
Doronicum pardalianches
Abrotanella trilobata
Mutisia decurrens
Carduus nutans
Wunderlichia mirabilis
Cichorium bottae
Barnadesia caryophylla
1,13
3,43
4,55
5,24
5,94
6,83
1,34
3,57
11,16
0,19
3,58
8,03
5,14
10,17
11,81
4,83
6,96
4,51
8,75
14,8
10,55
7,53
16,43
8,73
18,78
9,7
12
13,9
22,31
2,85
4,73
7,17
5,72
8,61
27,2
19,57
15,86
32,46
20,86
42,78
19,79
17,99
52,02
25,4
31,77
37,44
28,06
40,61
59,1
45,2
66,76
42,88
74,03
47,24
125
100
75
50
25
0

Figure S14

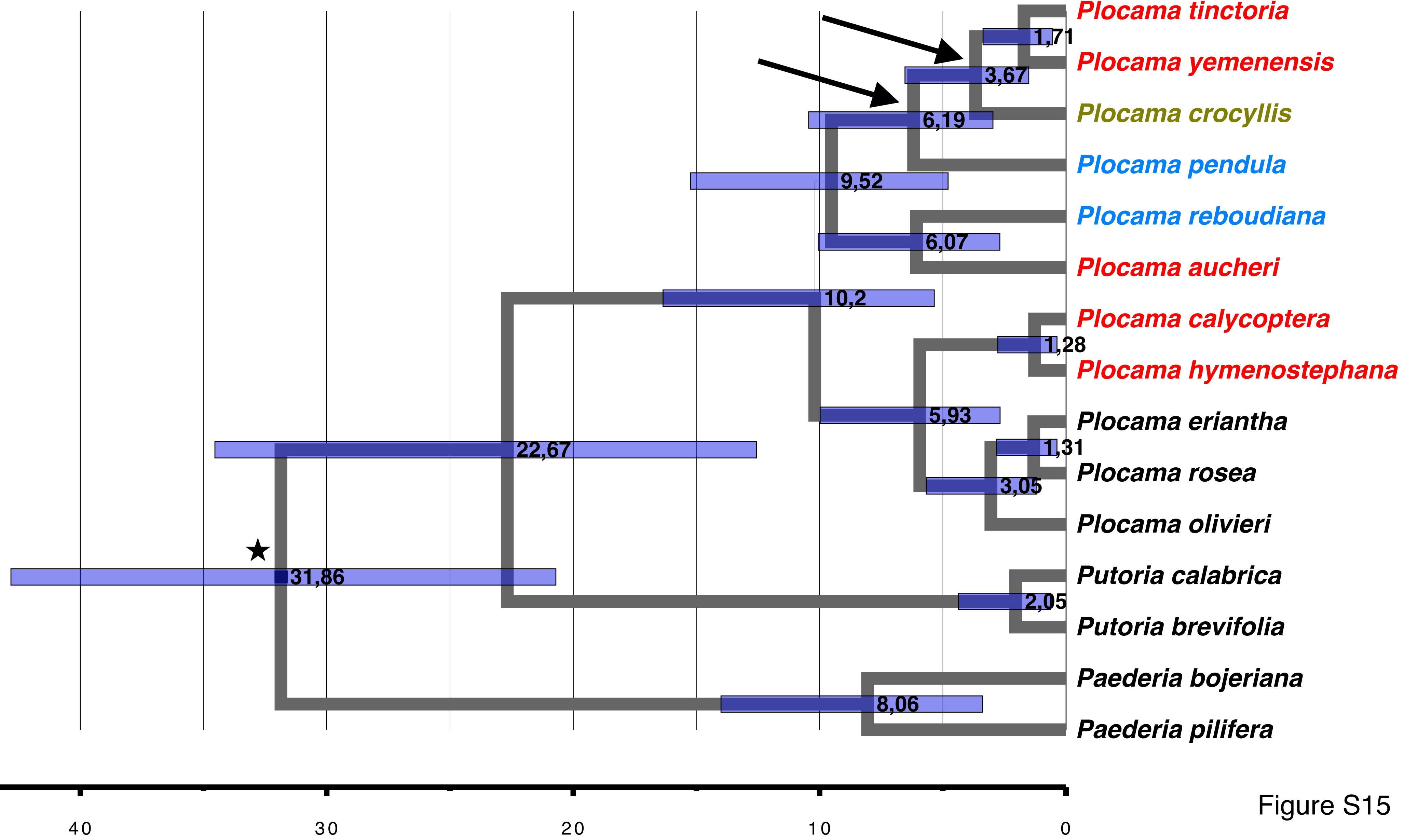
Plocama tinctoria
1,71
Plocama yemenensis
3,67
Plocama crocyllis
6,19
Plocama pendula
9,52
Plocama reboudiana
6,07
Plocama aucheri
10,2
Plocama calycoptera
1,28
Plocama hymenostephana
Plocama eriantha
5,93
22,67
1,31
Plocama rosea
3,05
Plocama olivieri
31,86
Putoria calabrica
2,05
Putoria brevifolia
Paederia bojeriana
8,06
Paederia pilifera
40
30
20
10
0

Figure S15

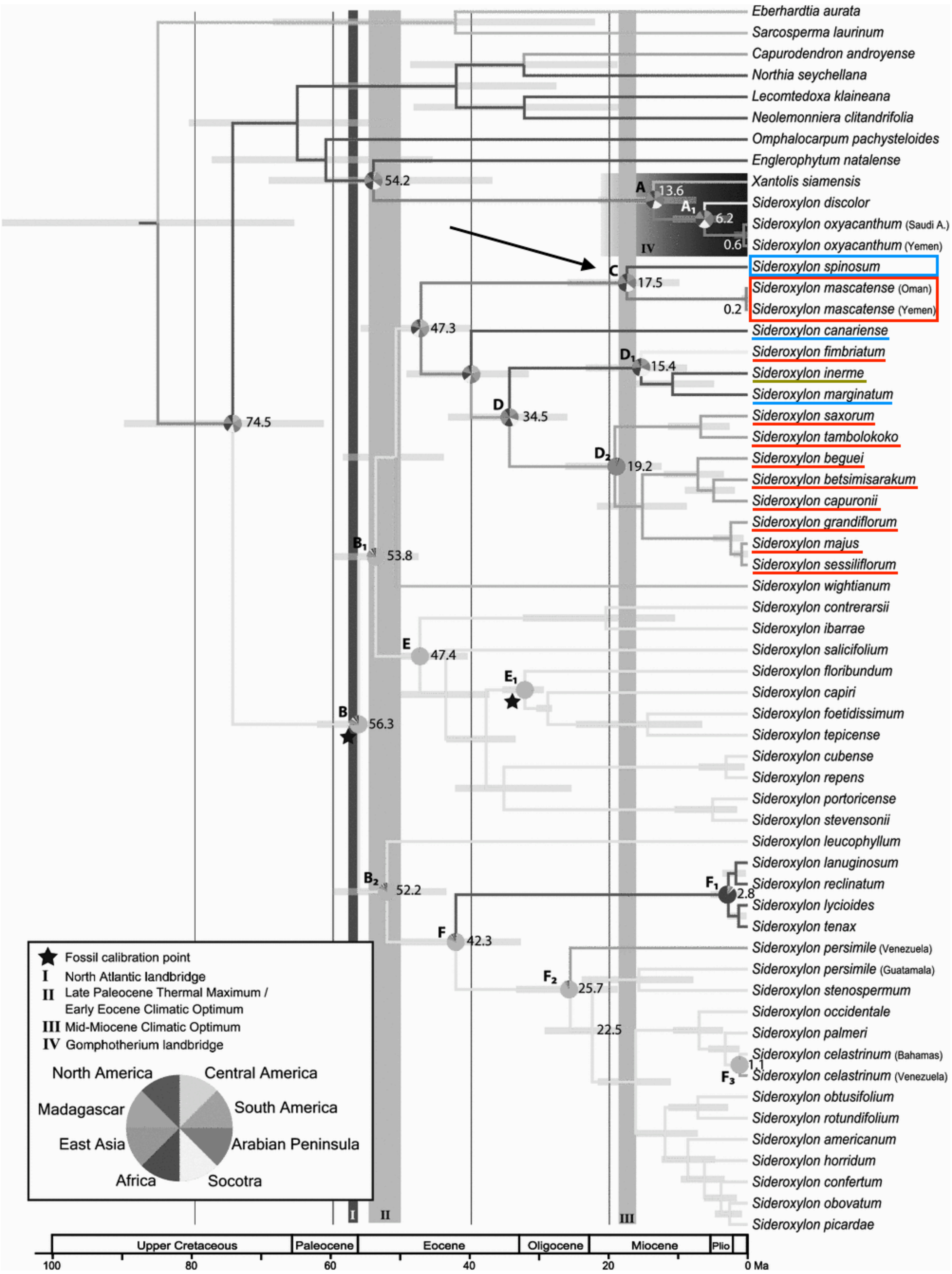

Eberhardtia aurata
Sarcosperma laurinum
Capurodendron androyense
Northia seychellana
Lecomtedoxa klaineana
Neolemonniera clitandrifolia
Omphalocarpum pachysteloides
Englerophytum natalense
Xantolis siamensis
Sideroxylon discolor
Sideroxylon oxyacanthum (Saudi A.)
Sideroxylon oxyacanthum (Yemen)
Sideroxylon spinosum
Sideroxylon mascatense (Oman)
Sideroxylon mascatense (Yemen)
Sideroxylon canariense
Sideroxylon fimbriatum
Sideroxylon inerme
Sideroxylon marginatum
Sideroxylon saxorum
Sideroxylon tambolokoko
Sideroxylon beguei
Sideroxylon betsimisarakum
Sideroxylon capuronii
Sideroxylon grandiflorum
Sideroxylon majus
Sideroxylon sessiliflorum
Sideroxylon wightianum
Sideroxylon contrerarsii
Sideroxylon ibarrae
Sideroxylon salicifolium
Sideroxylon floribundum
Sideroxylon capiri
Sideroxylon foetidissimum
Sideroxylon tepicense
Sideroxylon cubense
Sideroxylon repens
Sideroxylon portoricense
Sideroxylon stevensonii
Sideroxylon leucophyllum
Sideroxylon lanuginosum
Sideroxylon reclinatum
Sideroxylon lycioides
Sideroxylon tenax
Sideroxylon persimile (Venezuela)
Sideroxylon persimile (Guatamala)
Sideroxylon stenospermum
Sideroxylon occidentale
Sideroxylon palmeri
Sideroxylon celastrinum (Bahamas)
Sideroxylon celastrinum (Venezuela)
Sideroxylon obtusifolium
Sideroxylon rotundifolium
Sideroxylon americanum
Sideroxylon horridum
Sideroxylon confertum
Sideroxylon obovatum
Sideroxylon picardae
A 13.6
A1 6.2
0.6
IV
C 17.5
0.2
54.2
47.3
D1 15.4
D 34.5
D2 19.2
74.5
B1 53.8
E 47.4
E1
B 56.3
B2 52.2
F1 2.8
F 42.3
F2 25.7
22.5
1.1 F3
I
II
III
Fossil calibration point
I North Atlantic landbridge
II Late Paleocene Thermal Maximum / Early Eocene Climatic Optimum
III Mid-Miocene Climatic Optimum
IV Gomphotherium landbridge
North America
Central America
Madagascar
South America
East Asia
Arabian Peninsula
Africa
Socotra
Upper Cretaceous
Paleocene
Eocene
Oligocene
Miocene
Plio
100
80
60
40
20
0 Ma

Figure S16

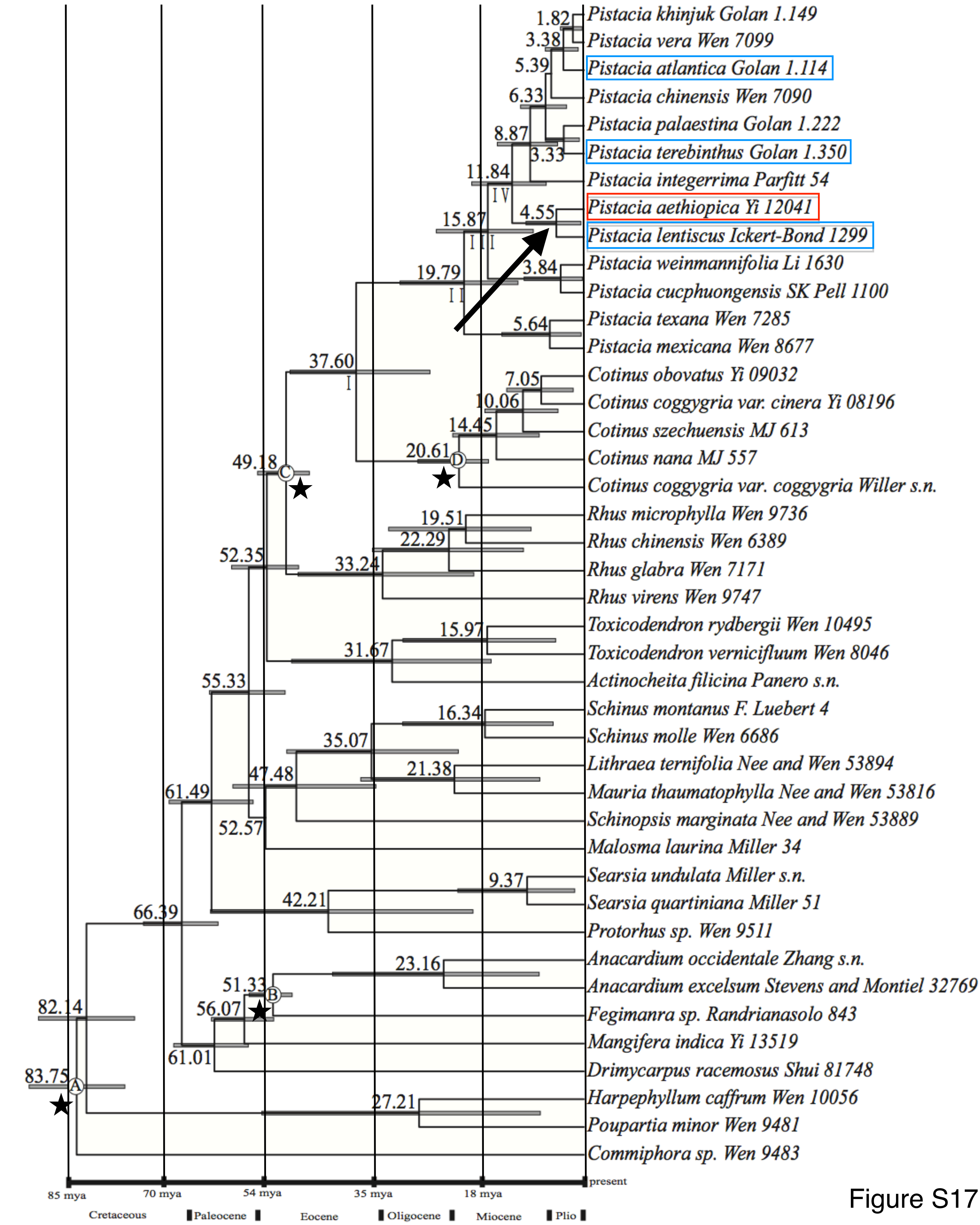
Pistacia khinjuk Golan 1.149
Pistacia vera Wen 7099
Pistacia atlantica Golan 1.114
Pistacia chinensis Wen 7090
Pistacia palaestina Golan 1.222
Pistacia terebinthus Golan 1.350
Pistacia integerrima Parfitt 54
Pistacia aethiopica Yi 12041
Pistacia lentiscus Ickert-Bond 1299
Pistacia weinmannifolia Li 1630
Pistacia cucphuongensis SK Pell 1100
Pistacia texana Wen 7285
Pistacia mexicana Wen 8677
Cotinus obovatus Yi 09032
Cotinus coggygria var. cinera Yi 08196
Cotinus szechuensis MJ 613
Cotinus nana MJ 557
Cotinus coggygria var. coggygria Willer s.n.
Rhus microphylla Wen 9736
Rhus chinensis Wen 6389
Rhus glabra Wen 7171
Rhus virens Wen 9747
Toxicodendron rydbergii Wen 10495
Toxicodendron vernicifluum Wen 8046
Actinocheita filicina Panero s.n.
Schinus montanus F. Luebert 4
Schinus molle Wen 6686
Lithraea ternifolia Nee and Wen 53894
Mauria thaumatophylla Nee and Wen 53816
Schinopsis marginata Nee and Wen 53889
Malosma laurina Miller 34
Searsia undulata Miller s.n.
Searsia quartiniana Miller 51
Protorhus sp. Wen 9511
Anacardium occidentale Zhang s.n.
Anacardium excelsum Stevens and Montiel 32769
Fegimanra sp. Randrianasolo 843
Mangifera indica Yi 13519
Drimycarpus racemosus Shui 81748
Harpephyllum caffrum Wen 10056
Poupartia minor Wen 9481
Commiphora sp. Wen 9483
1.82
3.38
5.39
6.33
8.87
3.33
11.84
IV
4.55
15.87
III
19.79
3.84
II
5.64
37.60
I
7.05
10.06
14.45
20.61
D
49.18
C
19.51
22.29
52.35
33.24
15.97
31.67
55.33
16.34
35.07
21.38
47.48
61.49
52.57
9.37
42.21
66.39
23.16
51.33
B
56.07
82.14
61.01
83.75
A
27.21
85 mya
70 mya
54 mya
35 mya
18 mya
present
Cretaceous
Paleocene
Eocene
Oligocene
Miocene
Plio

Figure S17
